# Earth history events shaped the evolution of uneven biodiversity across tropical moist forests

**DOI:** 10.1073/pnas.2026347118

**Published:** 2021-10-01

**Authors:** Oskar Hagen, Alexander Skeels, Renske E. Onstein, Walter Jetz, Loïc Pellissier

**Affiliations:** ^a^Landscape Ecology, Institute of Terrestrial Ecosystems, Department of Environmental Systems Science, Eidgenössische Technische Hochschule (ETH) Zürich, 8092 Zürich, Switzerland;; ^b^Unit of Land Change Science, Swiss Federal Research Institute for Forest, Snow and Landscape (WSL), 8903 Birmensdorf, Switzerland;; ^c^Evolution and Adaptation Research Group, German Centre for Integrative Biodiversity Research (iDiv) Halle–Jena–Leipzig, 04103 Leipzig, Germany;; ^d^Department of Ecology and Evolutionary Biology, Yale University, New Haven, CT 06520;; ^e^Center for Biodiversity and Global Change, Yale University, New Haven, CT 06520

**Keywords:** plate tectonics, paleoclimate, gen3sis, mechanistic modeling, pantropical diversity disparity

## Abstract

Far from a uniform band, the biodiversity found across Earth’s tropical moist forests varies widely between the high diversity of the Neotropics and Indomalaya and the relatively lower diversity of the Afrotropics. Explanations for this variation across different regions, the “pantropical diversity disparity” (PDD), remain contentious, due to difficulty teasing apart the effects of contemporary climate and paleoenvironmental history. Here, we assess the ubiquity of the PDD in over 150,000 species of terrestrial plants and vertebrates and investigate the relationship between the present-day climate and patterns of species richness. We then investigate the consequences of paleoenvironmental dynamics on the emergence of biodiversity gradients using a spatially explicit model of diversification coupled with paleoenvironmental and plate tectonic reconstructions. Contemporary climate is insufficient in explaining the PDD; instead, a simple model of diversification and temperature niche evolution coupled with paleoaridity constraints is successful in reproducing the variation in species richness and phylogenetic diversity seen repeatedly among plant and animal taxa, suggesting a prevalent role of paleoenvironmental dynamics in combination with niche conservatism. The model indicates that high biodiversity in Neotropical and Indomalayan moist forests is driven by complex macroevolutionary dynamics associated with mountain uplift. In contrast, lower diversity in Afrotropical forests is associated with lower speciation rates and higher extinction rates driven by sustained aridification over the Cenozoic. Our analyses provide a mechanistic understanding of the emergence of uneven diversity in tropical moist forests across 110 Ma of Earth’s history, highlighting the importance of deep-time paleoenvironmental legacies in determining biodiversity patterns.

## Significance

Tropical moist forests harbor much of the world’s biodiversity, but this diversity is not evenly distributed globally, with tropical moist forests in the Neotropics and Indomalaya generally exhibiting much greater diversity than in the Afrotropics. Here, we assess the ubiquity of this “pantropical diversity disparity” (PDD) using the present-day distributions of over 150,000 species of plants and animals, and we compare these distributions with a spatial model of diversification combined with reconstructions of plate tectonics, temperature, and aridity. Our study demonstrates that differences in paleoenvironmental dynamics between continents, including mountain building, aridification, and global temperature fluxes, can explain the PDD by shaping spatial and temporal patterns of species origination and extinction, providing a close match to observed distributions of plants and animals.

Tropical and subtropical moist broadleaf forests, including evergreen tropical rain forests and wet seasonal forests (hereafter tropical moist forests), are the most species-rich terrestrial biome on the planet (1–3) and are most broadly distributed throughout the Amazon basin and Atlantic forest in the Neotropics, the Congo basin and Rift Mountains in the Afrotropics, and both mainland and archipelagic South and Southeast Asia in Indomalaya (4). While all three major tropical moist forest regions (hereafter Neotropics, Afrotropics, and Indomalaya) have an exceptionally high species diversity of plants and animals in comparison with other biomes, the total regional diversity (γ-diversity) and number of species that coexist locally (α-diversity) vary dramatically across continents (2). Specifically, moist forests in the Afrotropics typically harbor lower species diversity than the Neotropics and Indomalaya (5–9), leading the Afrotropics to be labeled as the “odd man out” (9). We refer to this phenomenon as pantropical diversity disparity (PDD). This pattern has been highlighted in several keystone taxa, such as palms (family Arecaceae), which—of roughly 2,500 species globally—have ∼1,200 species in Indomalaya and 800 species in the Neotropics but only 66 species in the Afrotropics (excluding Madagascar) (10, 11). Investigating the drivers of variation in species diversity in moist forests across continents could provide an alternative perspective for understanding the processes that have shaped extraordinary tropical diversity.

Explanations for the PDD have been expressed in terms of both contemporary differences in carrying capacities between regions based on the distribution of key environmental variables (9, 12, 13) and historical differences in paleoenvironmental dynamics shaping the past distribution of tropical biomes (14–19) and patterns of diversification (20–22). Species diversity in tropical moist forests may be driven by contemporary climate conditions if energy and resource availability from high precipitation, temperature, and solar radiation facilitates a greater number of coexisting species (2, 23). These environmental features have been shown to explain significant variation in species diversity along a terrestrial latitudinal gradient (24), yet they also vary longitudinally between tropical regions (2) with, for example, the Afrotropics lacking analogous sites of aseasonal high precipitation found in the Neotropics and Indomalaya, which are among the most biodiverse in these regions (12). Tropical biomes in different regions also have had dramatically different paleoenvironmental histories, associated with distinct geological and climatic dynamics (2, 9, 14, 25), which may have driven variation in speciation, extinction, and dispersal rates between regions owing to dynamic patterns of fragmentation, connectivity, and habitat heterogeneity (25, 26). For example, previous paleoenvironmental reconstructions indicate that while moist forests in the Neotropics and Indomalaya have remained relatively constant in size since the Eocene, moist forests in the Afrotropics suffered a drastic reduction in area from the Miocene onward (14, 20), which is believed to have driven widespread extinction from range contractions (25, 27). Additionally, Afrotropical moist forests lay at the center of the African tectonic plate and therefore, lack the intersection of active orogeny at plate boundaries with terrestrial mesic equatorial habitat as seen in the Neotropical and Indomalayan regions, leading to the formation of the Andes in the Neotropics and the Himalayan and southwest Chinese mountain chains, as well as the Southeast Asian archipelago in Indomalaya (2, 15). Active orogeny has presented dynamic opportunities for ecological and allopatric speciation (19, 28, 29) and may explain the disparity in biodiversity among tropical regions.

Drawing inferences about historical processes that have shaped the PDD has been challenging and restricted by the limited mechanistic understanding of ecological and evolutionary processes from correlative or comparative methods (30). Instead, by combining paleoenvironmental reconstructions with spatially explicit models of ecoevolutionary processes, simulation models offer a unique but largely underused resource (but see, for example, refs. 31–34) to directly explore the evolutionary mechanisms behind the origins of biodiversity patterns in silico (30, 34). In this study, we explored the origins of the PDD in three steps. First, we quantified the ubiquity of the PDD across a wide range of plant and animal taxa. We then tested whether contemporary climate conditions can explain variation in species diversity among continents using a correlative approach. Finally and most innovatively, we assessed the role of paleoenvironmental dynamics in driving pantropical biodiversity patterns using a spatially explicit simulation model of diversification coupled with a paleoenvironmental reconstruction of temperature, aridity, and plate tectonics over the past 110 Ma. Specifically, we explored how major changes in the paleoclimate and plate tectonics have shaped speciation and extinction rates throughout the Mesozoic and Cenozoic and the spatial distribution of phylogenetic diversity. We asked the following questions. 1) Are present-day climatic differences between continents sufficient to explain differences in species diversity? 2) Could deep-time environmental dynamics have driven the emergence of present-day diversity differences between regions? 3) How has spatial and temporal variation in speciation and extinction rates shaped spatial diversity patterns, and how have mountain building, island formation, global cooling, and aridification influenced these rates? 4) What is the deep-time signature of diversification and dispersal in spatial patterns of phylogenetic diversity?

## Results and Discussion

### Contemporary Variation in Biodiversity and Climate between Tropical Moist Forests.

**Fig. 1.**
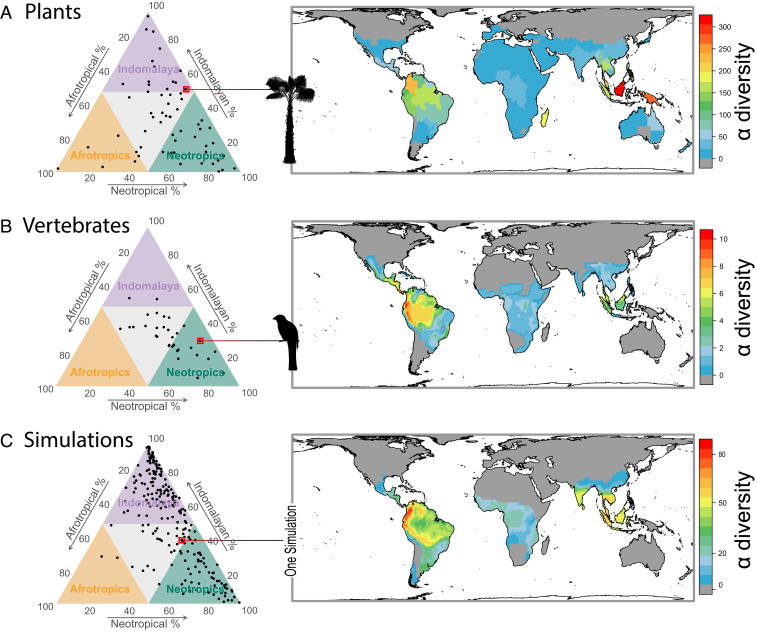
Evenness of diversity in tropical moist forests across biogeographic regions in pantropically distributed taxa. Ternary plots show the proportions of diversity per clade found in Neotropical, Afrotropical, and Indomalayan tropical moist forests (green, orange, and purple triangles, respectively) for (*A*, *Left*) plant families; (*B*, *Left*) mammal, bird, and amphibian orders and squamate infraorders; and (*C*, *Left*) mechanistic model simulations. Species richness maps highlight examples that show the PDD: (*A*, *Right*) Arecaceae (palms; richness measured across botanical countries), (*B*, *Right*) Trogoniformes (trogons and allies), and (*C*, *Right*) one simulation.

We assessed the ubiquity of the PDD pattern across a broad range of taxa using distribution data for over 128,000 species of plants from 165 families (35) and over 32,000 species of terrestrial vertebrates from 71 bird, mammal, and amphibian orders and 7 squamate reptile infraorders (36–38). We found that 38 vertebrate and 63 plant clades, encompassing 95% of all terrestrial vertebrate species and 92% of all plant species assessed, are distributed across tropical moist forests in all three of the investigated regions: Neotropical, Afrotropical, and Indomalayan (3). For these pantropical taxa, we found a systematic pattern of lower γ-diversity in the Afrotropics, with 23 vertebrate clades and 34 plant clades—representing 81 and 72% of all vertebrate and plant species, respectively—showing a PDD pattern (Fig. 1 *A* and *B* and Dataset S1). This result highlights that a disproportionate number of species belong to large tropical radiations, including anuran amphibians, passerine birds, gekkotan squamates, chiropteran mammals, and orchids, and these ecologically distinct but extraordinarily diverse clades show strikingly similar uneven diversity across the tropics.

**Fig. 2.**
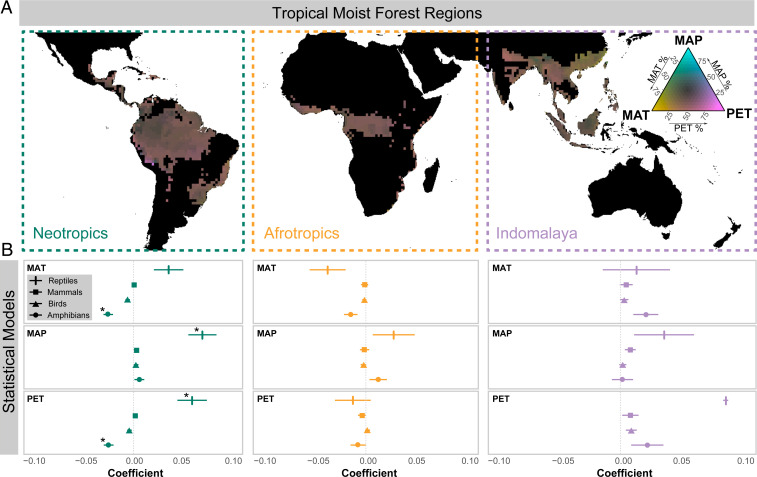
Present-day environmental variation among tropical moist forest regions and species richness relationships. (*A*) Ternary color-coded map of tropical moist forest regions based on PET (955.4, 1,953.6 mm/y), MAP (389.8, 6,527.3 mm/y), and MAT (5.3, 28.5 °C). (*B*) Regression coefficients from GLS models of log(species richness + 1) as a function of PET, MAP, and MAT for four vertebrate clades (squamate reptiles, mammals, birds, and amphibians) and three regions (Neotropics, Afrotropics, and Indomalaya), with each clade and region tested separately. *Statistical significance after Holm’s correction for multiple comparisons.

To explore whether variation in contemporary climate might explain the PDD, we compared the distribution of mean annual temperature (MAT), mean annual precipitation (MAP), and annual potential evapotranspiration (PET) in the present day across each of the three tropical moist forest regions. Our results support the assertion that the Afrotropics contain only a subset of the total environmental variation present in the Neotropical and Indomalayan regions (12), corresponding to an absence of regions with very high MAP (>3,300 mm) and very low MAT (<13°C) (Fig. 2*A* and *SI Appendix*, Figs. S1 and S2). It has been suggested that high-precipitation environments in the Neotropics and Indomalaya, with no analog in the Afrotropics, are among the world’s most species rich (12). The effects of temperature and precipitation on species diversity have been suspected to be the indirect result of their positive influence on primary productivity (23). However, we found that both median and maximum PET, a measure of productivity, were actually highest in the Afrotropics, where diversity was lowest (*SI Appendix*, Fig. S1).

We quantified the relationships between α-diversity, MAT, MAP, and PET in 110- × 110-km grid cells across tropical moist forest regions in four vertebrate clades for which high-resolution spatial data were available (amphibians, squamate reptiles, mammals, and birds) using generalized least squares (GLS) models accounting for spatial autocorrelation (Fig. 2*B*), and we found only weak support for a general relationship between richness and climate. Of the 36 relationships tested, only 4 relationships were significant after correcting for multiple comparisons (*P* < 0.05; Holm’s correction) (*SI Appendix*, Table S2), and these significant relationships had a small effect size (Fig. 2*B*). For example, a 400-mm gradient of MAP within the Neotropics predicted a difference of ∼2 squamate species, while that same difference, as found on average between moist forests of the Neotropics and Afrotropics, was associated with an average difference of 18 species between the continents. Our results are also contrary to findings from several studies on plant diversity (12, 39, 40), bringing into question the generality of a present-day climate–richness relationship across taxa and spatial scales within tropical moist forests and the role of contemporary climate as the primary driver of tropical diversity.

### Simulated Variation in Biodiversity between Tropical Moist Forests.

To investigate how paleoenvironmental dynamics have shaped present-day patterns of biodiversity across tropical moist forests, we implemented a spatially explicit process-based simulation model of diversification (34) using global paleoenvironmental reconstructions of temperature and aridity dynamics from the Mid-Cretaceous (41). We ran the simulation model 500 times, starting with a single ancestral species distributed throughout the equatorial tropics at 110 Ma. We explored a scenario of pantropical origination corresponding to the ancestors of tropical lineages originating around 120 to 100 Ma (42, 43), before radiating substantially following the Cretaceous–Paleogene boundary (44–46). This period also coincides with the splitting of the American and African continents, opening the Equatorial Atlantic Ocean, and the formation of modern megathermal moist forest ecosystems (16, 47). Our model, implementing only a parsimonious set of ecoevolutionary processes (dispersal, environmental filtering, environmental niche evolution, and speciation) and one additional constraint on dispersal into arid sites, shows that plate tectonics coupled with changes in paleotemperature and aridity can help explain the systematic variation in species diversity across tropical moist forests.

The model reconstructed a range of tropical biodiversity patterns across tropical regions and along latitude. Of the 500 simulations, 106 resulted in total extinction or generated diversity greater than 12,500 species, a threshold beyond which simulations become computationally intractable. The remaining 394 simulations generated a gradient of diversity with latitude, with species richness being negatively correlated with absolute latitude (mean Spearman’s ρ = −0.60, range = [−0.76, −0.12]). Furthermore, 221 of the complete simulations (56%) generated pantropical diversity, and 169 of these (42%) generated the PDD pattern seen in the empirical data (Fig. 1*C* and *SI Appendix*, Fig. S3). To investigate the sensitivity of broad-scale patterns of species distributions to the model parameters, we fitted generalized linear models with a binomial link function of a pantropical index (whether a simulation generated diversity in all three tropical regions) and a pantropical disparity index (whether simulations generated lower diversity in Afrotropical than in Neotropical or Indomalayan regions—the PDD) and model parameters. We found that when rates of temperature niche evolution were high, temperature niche widths were narrow, and when the speciation threshold was high, simulations were less likely to generate pantropical diversity (*SI Appendix*, Table S4) or the PDD (*SI Appendix*, Table S5). Under these parameters, lineages rapidly evolve unsuitable thermal tolerances, driving range collapses from mismatches with the environment. This resulted in extinction events outpacing speciation events, leading to total extinctions in the Afrotropics (67 simulations) and Indomalaya (163 simulations) and preventing the establishment of a pantropical distribution. Instead, simulations with stricter niche conservatism (σ < 0.01) regularly generated lineages present in all three tropical regions and a strong PDD (169 of 221 pantropical simulations), as species with low rates of thermal niche evolution maintained adaptation to the megathermal environment through the relatively stable temperature changes of the Cenozoic in the equatorial tropics (48, 49). This result reinforces other recent simulation studies, which also found that niche conservatism is instrumental in generating realistic biodiversity gradients (32, 33).

### Empirical and Simulated Species Richness Patterns.

We found that species richness patterns across simulation outputs were positively correlated with observed richness patterns for vertebrate and plant taxa distributed in all tropical regions (Fig. 1). The highest recorded Spearman correlation coefficients between species richness across 110- × 110-km grid cells for each vertebrate clade and completed simulations ranged from 0.49 to 0.91, with a median of 0.78 (*n* = 22), and correlation coefficients for species richness summarized within botanical countries for plant families ranged from 0.25 to 0.82, with a median of 0.61 (*n* = 34). Very few clades had correlation coefficients of <0.5, and those that did typically had idiosyncratic diversity patterns that did not match the more general features of the PDD. For example, the Anguimorpha (monitor lizards and allies) showed a PDD with only 3 species found in the moist forests of the Afrotropics, compared with 26 and 91 in Indomalaya and the Neotropics, respectively. However, Neotropical hot spots of diversity for the Anguimorpha are located in Central America with the clade being almost entirely absent from the Amazon basin, contrary to the simulation model predictions (Fig. 1*C*). This suggests that the simulation model can capture general features of pantropical diversity, while the biogeography of individual taxa requires further examination.

Previous correlative models incorporating historical variation in area and productivity have also provided a close fit to global patterns of species richness (14, 18, 50), yet these models have not been explicitly evolutionary and have been unable to explicitly investigate macroevolutionary processes shaping biodiversity patterns. On the other hand, similar mechanistic models have been used to explore the emergence of terrestrial diversity gradients (32, 33), although the origin of the PDD has not been investigated in previous studies. For example, Rangel et al. (33) looked exclusively at the evolution of Neotropical diversity, while the global study of Saupe et al. (32), which considered paleoenvironmental change over the past 120 ky, successfully reconstructed the latitudinal diversity gradient but overpredicted diversity in Afrotropical moist forests; therefore, it did not capture the PDD. The inclusion of extended paleoenvironmental reconstructions from the Mesozoic (110 Ma) to the present day (41, 49, 51) in the present study may explain why the model was able to additionally predict pantropical diversity patterns.

### Paleoenvironmental Dynamics and Macroevolutionary Rates in Tropical Moist Forests.

**Fig. 3.**
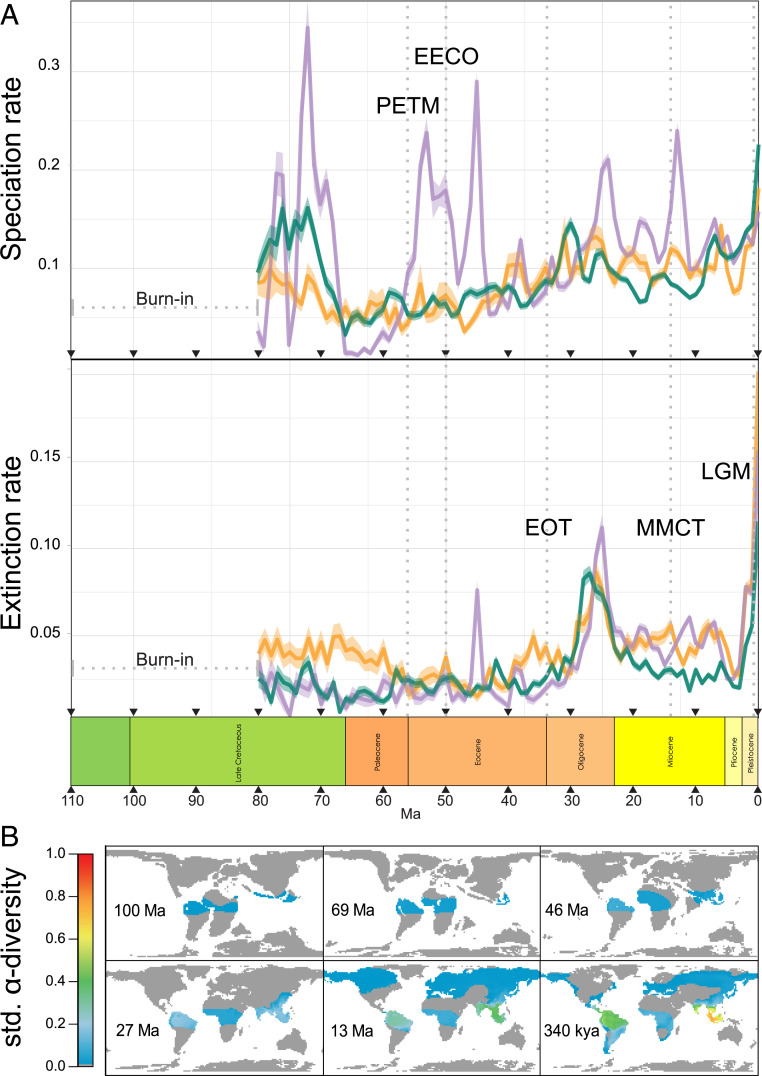
Macroevolutionary rates and spatial diversity patterns through time from simulation models. (*A*) Speciation and extinction rates estimated across 1-My intervals within each tropical moist forest region, averaged across models that generated the PDD. Rates were highly stochastic in the Mid-Cretaceous (burn-in period) because species diversity was low, so we present rates from the Late Cretaceous (80 Ma) onward. Several key climatic periods are highlighted with dashed gray lines. The greenhouse climates of the Paleocene and Eocene are highlighted by the Paleocene–Eocene thermal optimum (PETM; ∼56 Ma) and the Early Eocene climatic optimum (EECO; ∼50 Ma), while global cooling and the transition to an icehouse climate are highlighted by the Eocene–Oligocene transition (EOT; ∼34 Ma), the Mid-Miocene climate transition (MMCT; ∼14 Ma), and the last glacial maximum (LGM; ∼30 to 10 ka). (*B*) Species richness patterns standardized and averaged across simulations generating the PDD during six time periods corresponding to the Mid-Cretaceous (100 Ma), Late Cretaceous (69 Ma), Eocene (46 Ma), Oligocene (27 Ma), Miocene (13 Ma), and Pleistocene (340 ka).

To investigate the macroevolutionary dynamics that lead to the diversity differences between the tropical moist forest regions, we extracted spatial and temporal variation in speciation and extinction rates across the subset of simulations that generated the PDD (169 simulations) (Fig. 3). We performed a pairwise comparison of the distribution of mean speciation and extinction rates between regions using Wilcoxon signed-rank tests and found that Indomalaya, but not the Neotropics, had significantly higher rates of speciation compared with the Afrotropics (Neotropics mean = 0.089 ±0.002 speciation events per lineage per My, Indomalaya = 0.117 ±0.001, Afrotropics = 0.083±0.002), although both the Neotropics and Indomalaya had significantly lower rates of extinction than the Afrotropics (Neotropics = 0.029 ±0.001 extinction events per lineage per My, Indomalaya = 0.033 ±0.001, Afrotropics = 0.042 ±0.002). Our findings unify two alternative hypotheses (2): that both lower speciation rates in the Afrotropics (21, 52) and higher extinction rates in the Afrotropics (15, 25) compared with the other moist forest regions have played a role in shaping the PDD. Moreover, both speciation and extinction rates were significantly higher in Indomalaya than in the Neotropics, resulting in overall higher species turnover, and as such, tropical moist forests in the Neotropics and Indomalaya reached high diversity through alternative pathways.

#### Aridification.

The simulation results support the long-term role of aridification in shaping contemporary Afrotropical biodiversity by increasing extinction through range contractions and decreasing the area of opportunity for speciation (16). Presently, Afrotropical moist forests occupy the smallest geographic area of the three regions (2) and are tightly bound by the Sahara, Namib, and Ogaden deserts, but the Afrotropics are considered to have had the largest area of moist forests of any continent during the “greenhouse” climates of the Paleocene and Eocene (14, 20, 47) (*SI Appendix*, Fig. S4). A subsequent decrease in area may have begun as early as the Mid-Eocene (25, 53), and it became more pronounced due to rapid global cooling at the Eocene–Oligocene transition and later during the Middle Miocene climate transition and Late Miocene cooling events (16, 47, 54). Along with simultaneous tectonic rifting activities, these changes altered the distribution of precipitation across the African continent (47, 55, 56). A number of phylogenetic studies have suggested deep divergences between moist forest lineages in eastern and western African dating as early as the Eocene and Oligocene (57, 58), driven by the ancient vicariance of these regions following the expansion of dry habitats (59). According to the simulations, the reduction in the extent of moist forests over time has had legacy effects on the biodiversity of taxa associated with tropical moist forests (2). Specifically, extinction rates were highest in the Afrotropics during the Paleocene and Eocene (Neotropics Paleocene = 0.019 ±0.0009, Eocene = 0.023 ±0.0005; Indomalaya Paleocene = 0.018 ±0.001, Eocene = 0.021 ±0.0007; Afrotropics Paleocene = 0.032 ±0.0018; Eocene = 0.028 ±0.0009), while differences compared with other regions were less marked during the Miocene (Neotropics Miocene = 0.019 ±0.0009, Indomalaya Miocene = 0.046 ±0.0005, Afrotropics Miocene = 0.046 ±0.0009) (Fig. 3 and *SI Appendix*, Fig. S5).

To assess the causal role of key Earth history changes on emergent biodiversity patterns, we ran simulations that modified the paleoenvironmental reconstructions by removing the aridity constraint in the Afrotropical realm from the Early Cretaceous and from the Early Miocene. These supporting simulations showed that lifting aridity constraints in the Afrotropics from the Miocene onward did not significantly influence diversity (paired Wilcoxon signed-rank test *P* = 0.07), while simulations with the aridity constraint removed from the Early Cretaceous showed significantly higher diversity than unmodified simulations (original simulations generated on average 44.8% of the diversity of modified simulations; Wilcoxon test *P* = 0.027), reversing the PDD in ∼66% of simulations (*SI Appendix*, Fig. S6). In particular, when the constraint of aridity was removed from the Cretaceous, diversification rates were higher, driven by the opening up of a large area for lineages to radiate, including many of the southern and northern parts of the continent (16, 49). Our results, based on reconstructions of paleoaridity dynamics, suggest that aridification played an early and sustained role in suppressing Afrotropical moist forest diversity over a long period of the Cretaceous and Cenozoic.

#### Plate tectonics and orogenesis.

A strong association between plate tectonics and the formation of uneven tropical diversity across regions emerged from the simulations, with the rise of the Andes playing a key role in the Neotropics. Dynamic tectonic activity has been suggested to be the cause of exceptional biodiversity in both the Neotropics and Indomalaya (15, 19) as both these regions have active continental margins compared with the Afrotropics, which lies at the center of the African plate and whose major topographic features are the result of rifting (47, 56). The formation of the Andes, resulting from plate convergence between the South American continent and the subducting oceanic Nazca plate, has been proposed to foster lineage diversification, acting as a source of diversity across the Neotropics (19, 60). Accordingly, our simulations showed increasing rates of diversification from the Paleocene to the Oligocene in the Neotropics (Fig. 3 and *SI Appendix*, Fig. S6) associated with the early rise of the Andes in the paleoreconstruction and the associated increase in environmental heterogeneity (*SI Appendix*, Fig. S7). To further demonstrate the role of the Andes in forming Neotropical diversity, following ref. 33, we ran simulations in which Andean orogenesis was removed, holding a constant low elevation from 110 Ma onward. We found that removing orogeny led to significantly lower Neotropical diversity (modified simulations generated on average 1.6% of the diversity of the original simulations; paired Wilcoxon signed-rank text *P* < 0.001), reversing the PDD in more than 90% of the modified simulations (*SI Appendix*, Fig. S6).

The Indomalayan realm, in addition to the Neotropics, is both one of the Earth’s most biodiverse and tectonically active regions. The collision of the Indian and Eurasian plates led to the formation of some of the world’s largest mountain chains, promoting speciation in South and Southeast Asia, and may be the ultimate driver of several biodiversity hot spots (61–63). The collision of the Australian and Eurasian plates in the Early to Mid-Miocene led to the formation of the topographically complex Southeast Asian archipelago and—combined with dynamic sea-level changes—may have led to the origin of locally endemic biotas on intermittently isolated islands (64, 65). In the simulations, increasing rates of diversification in Indomalaya from the onset of the Indian–Eurasian plate collision in the Late Eocene until the Late Oligocene (∼25 Ma) suggest a key role of habitat complexity in shaping the diversification of this region (Fig. 3). Furthermore, high rates of speciation during the collision of the Australian and Eurasian plates in the Mid-Miocene also point to the role of either increased topographic complexity or island formation as key factors shaping the biodiversity (66). To investigate the origins of Indomalayan biodiversity and tease apart the effects of island isolation and mountain building, we ran simulations with modified landscapes, removing the cost of dispersal over water, as well as environmental heterogeneity following orogenesis. While reducing island isolation did not change diversity (Wilcoxon test *P* = 0.92), reducing heterogeneity caused significantly lower diversity (modified simulations generated on average 12.9% of the diversity seen in the original simulations; Wilcoxon test *P* = 0.002), reversing the PDD in >80% of the modified simulations (*SI Appendix*, Fig. S6). The role of isolation, while not integral to the establishment of high Indomalayan biodiversity in the simulations, has been shown to be important in clades with low dispersal capacities (67). We did not model variation in dispersal traits in this study; however, this may help to explain residual variation in biodiversity across lineages.

Taken together, variation in rates of diversification in the simulation models and the landscape modification experiments suggests a predominant role of active mountain building in the formation of the PDD. The Afrotropics are as topographically heterogeneous as the Indomalayan and Neotropical regions, with several major topographical features intersecting the tropical moist forest biome—including the Central African Rise, Cameroon Highlands, and Eastern Arc (2, 47, 68), some of which are centers of species richness (69). However, in the Afrotropics, mountain topography was generally more stable over time and associated with more arid continental regions (2). Hence, our results show that the dynamic temperature heterogeneity provided by active mountain building in mesic regions creates a mosaic of opportunities for species to adapt to different conditions, leading to high rates of diversification that act not only as cradles of diversification but also, as museums, refugia, and innovation hubs (70).

### Global Temperature Changes.

Speciation and extinction rates through time in tropical moist forests show complex patterns associated with both region-specific dynamics and global temporal trends in paleoenvironmental conditions. In the simulation model, speciation was dictated by the establishment of geographic isolation driven by temperature, aridity, or oceanic barriers, and as a result, idiosyncratic speciation dynamics (Fig. 3) were more strongly determined by local geoclimatic histories. On the other hand, extinction in the model was based on mismatches between species temperature niches and the landscape, and therefore, climate change was the primary driver of extinction, which might explain congruent trends in extinction dynamics resulting from global climate change over the past 110 Ma. Our simulations showed that the Late Paleogene period was characterized by increasing extinction rates, punctuated by a burst in extinction rates associated with ∼10 My of cooling climate at the Eocene–Oligocene transition (the “big chill”) (*SI Appendix*, Fig. S4), with extinction rates reaching a peak across all three regions in the Mid-Oligocene (Fig. 3 and *SI Appendix*, Fig. S5) (Oligocene average extinction rate in Afrotropics = 0.051 ±0.001, Indomalaya = 0.052 ±0.001, Neotropics = 0.055 ±0.001). Global cooling across the Eocene–Oligocene transition corresponds empirically to one of the Paleogene’s climate-driven global extinction events (71), which saw high rates of turnover in many taxa, including marine mollusks, tropical broadleaf plants, and terrestrial mammals (72–74). Further, fossil evidence has highlighted large decreases in diversity in Neotropical and Afrotropical forest biomes at this time (16).

A second shared extinction peak of a greater magnitude was recorded during the Late Neogene and Quaternary periods (Fig. 3 and *SI Appendix*, Fig. S5) (Pleistocene Afrotropical extinction rate = 0.119 ± 0.004, Indomalaya = 0.102 ±0.003, Neotropics = 0.072 ±0.003), resulting from global cooling and temperature oscillations associated with glaciation events. During these periods in the simulations, sharp changes in temperature (*SI Appendix*, Fig. S4) led to extinctions biased toward taxa at the upper and lower tails of the thermal niche distribution on each continent (*SI Appendix*, Fig. S8). This pattern was more severe in the Afrotropics and Indomalaya, as these regions had fewer species with thermal niches in the cold tails of the distribution and more species in the warm tails of the distribution (*SI Appendix*, Fig. S8). A possible explanation for this finding is that, while the Afrotropics are colder on average than the Neotropics or Indomalaya, the region had lower temperature heterogeneity overall and therefore, far fewer opportunities for species to adapt to the more extreme temperatures that increased in area during the Quaternary. Further, the large number of species in the warm tails of the thermal niche distribution in both Indomalaya and the Afrotropics were driven extinct by rapidly cooling climates (*SI Appendix*, Figs. S4, S8, and S9).

Temperature changes, such as those associated with the Eocene–Oligocene transition and Quaternary glaciations, drive range contractions due to a mismatch between thermal tolerances and available habitat, and in some cases, they drive total extinction of the species. Such temperature changes have been hypothesized to be a major driver of extinctions in tropical moist forests (11). However, in other cases where refugia exist, species are buffered from extinction (75), and temperature changes facilitate fragmentation of populations or sustain the persistence of already fragmented populations, leading to speciation (76). Our results show that both periods of high extinction coincided with high rates of speciation across all continents (Oligocene Afrotropical speciation rate = 0.106 ± 0.003, Indomalayan = 0.128 ± 0.002, Neotropical = 0.106 ± 0.002; Pleistocene Afrotropical speciation rate = 0.141 ± 0.004, Indomalayan = 0.138 ± 0.002, Neotropical = 0.168 ± 0.004) (Fig. 3 and *SI Appendix*, Fig. S5). These results together highlight the processes behind the dual role of climate change in diversification, where heterogeneous landscapes such as mountains act as both species pumps and refugia, as has been suggested for many Andean lineages (7, 19, 22, 28, 77–79). The role of glacial oscillations in generating species-level diversity, compared with intraspecific patterns of genetic divergence, is still debated, with population-level studies and phylogenetic studies showing evidence for both (e.g., refs. 80 and 81). In our simulations, the duration of speciation was parameterized to be longer than the timescales considered during the Pleistocene period, and so, the formation of new species in that era was due to the sustained isolation of lineages that began diverging before the Pleistocene.

### Paleoenvironmental Change and the Distribution of Phylogenetic Diversity in Tropical Moist Forests.

**Fig. 4.**
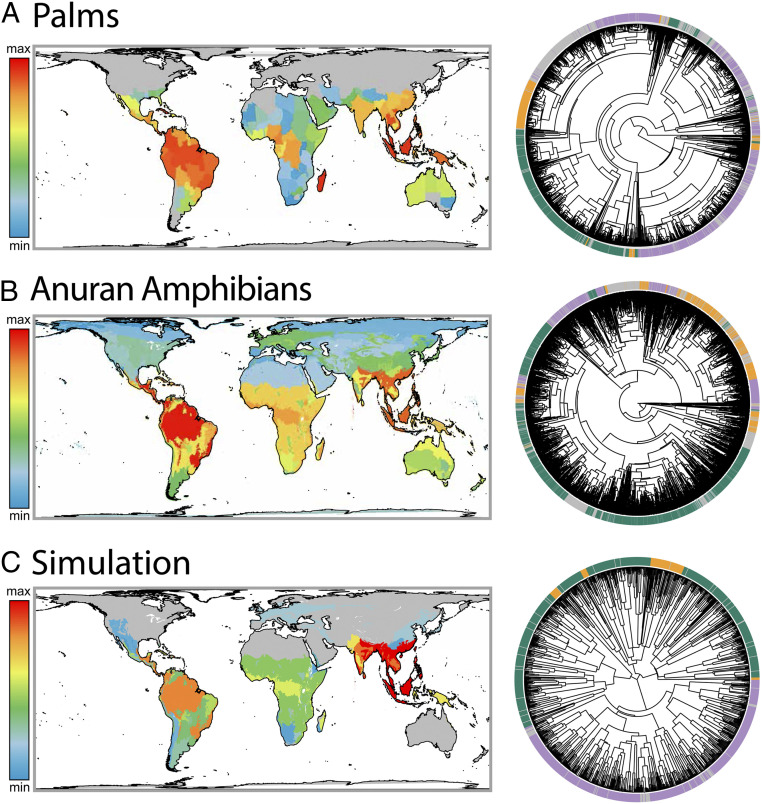
NRI measured across (*A*) palms (family Arecaceae; −9.6, 0.8) in botanical provinces, (*B*) anuran amphibians (−16.8, 0.1) in biomes, and (*C*) a gen3sis simulation (−40.4, 0.5) matching Figs. 1*C* and 3*B*, alongside tropical moist forest (TMF) biomes mapped onto associated phylogenies. Gray, non-TMF; green, Neotropics ; purple, Indomalaya; yellow, Afrotropics.

The phylogenetic structure of regional assemblages contains the signature of both the dispersal history and diversification dynamics that have shaped biodiversity patterns across regions (20, 82, 83). Using the net relatedness index [NRI (84)], we measured the degree to which species within tropical moist forests in different regions were more closely related (phylogenetic clustering) or more distantly related (phylogenetic overdispersion) than expected based on random sampling of species. In vertebrate clades showing the PDD pattern, we found support for significant phylogenetic clustering in 56% of clades in the Afrotropics, 61% in Indomalaya, and 74% in the Neotropics, compared with only 4% of clades in the Neotropics and Indomalaya that showed significant overdispersion and 8% of clades in the Afrotropics. The Afrotropics and Indomalaya had 34% of clades that showed distributions not significantly different from random, compared with 21% in the Neotropics, and the Neotropics on average were more phylogenetically clustered (*SI Appendix*, Fig. S10). This supports the assertion that tropical moist forests are generally composed of in situ endemic radiations (Fig. 4 *A* and *B*), which is consistent with an “out-of-the-tropics” model of diversification (82, 83, 85) rather than dispersal from other biomes, and also highlights the role of isolation of the Neotropics in establishing a highly clustered biota (20). However, contrary to findings from a study on palms (20), we observed that palms in the Afrotropics were not more likely to be randomly distributed than those in Indomalaya, and we found that, at the level of botanical country, palms were phylogenetically clustered in the Afrotropics (Fig. 4). This result supports the finding that phylogenetic clustering is increasingly common at larger spatial scales, due to the capturing of in situ diversification dynamics (86).

As a final evaluation of the validity of the simulation model, we estimated the ability of the model to reproduce NRI patterns in simulations showing a PDD. In our simulations, we found that all three tropical moist forest regions were significantly phylogenetically clustered in more than 95% of cases. More frequently, we found that tropical moist forests in Indomalaya and the Neotropics were more phylogenetically clustered than in the Afrotropics, similar to the empirical trend, and Spearman correlations between simulated and empirical NRIs were generally high (Spearman’s ρ = 0.22 to 0.89, median = 0.72) (Fig. 4). However, contrary to the empirical data, we found that Indomalayan assemblages were generally more clustered than those in the Neotropics (*SI Appendix*, Fig. S9). We attribute this pattern in the simulated data to the connection of the Afrotropics and Neotropics during the initial conditions of the simulation and to the isolation of the Indomalayan moist forests during much of the Cenozoic due to harsh aridity barriers to the north of the region, preventing dispersal into other biogeographic regions. However, empirically determined dispersal tracks between Indomalaya and the Palearctic, Afrotropics, and Australasia are widely recognized (15, 25), and many taxa originated after the split of the American and African continents. Investigating the role of long-distance dispersal in establishing patterns of phylogenetic diversity in a process-based simulation framework would be useful for understanding the relative contribution of in situ vs. dispersal-based processes in driving the PDD and other tropical biodiversity patterns.

## Conclusion

This study shows how paleoenvironmental change over the Mesozoic and Cenozoic has shaped variation in species diversity across tropical moist forests. Taken together, our results highlight the difficulty in assigning a proximal cause to any single process in shaping the PDD and instead, highlight the complex roles of habitat heterogeneity, aridity constraints, and temperature changes, as well as the importance of specific events in Earth’s history, in shaping global biodiversity patterns. We demonstrate that the origin of the PDD predates the Eocene–Oligocene transition (Fig. 3), supporting a deep history of the pattern (27, 87). However, the stark contrasts between tropical regions consistent with the PDD pattern we observe today were reinforced and consolidated during the Miocene, when extinctions rates were low in the Neotropics and speciation rates were high in Indomalaya relative to the Afrotropics. These contrasts further solidified during the Pleistocene, supporting an additional, but not ultimate, role of glacial oscillations. The model used in this study (34) can be applied to test hypotheses about spatial diversification dynamics and makes it possible to directly manipulate key Earth history events to better understand how biodiversity patterns emerge, a feature hard to achieve with currently available correlative or phylogenetic comparative methods.

Using only a simple set of ecoevolutionary rules played out across a dynamic landscape, we have demonstrated that we are able to reproduce emerging biodiversity patterns under a set of parameter combinations, particularly when thermal niches are phylogenetically conserved. The ability to reconstruct uneven tropical diversity highlights how simulation-based analyses can be used to explore different hypotheses of the processes shaping biodiversity gradients around the globe. In this study, we explored a single model of diversification and niche evolution, excluding a range of complex ecological processes, such as direct interspecific competition, which may explain residual variation between the simulated and empirical diversity patterns. We also applied a subset of parsimonious and generalized initialization and dispersal scenarios to taxa with a diverse range of biogeographic and evolutionary histories. We consider an important next step to be investigations of how adding or subtracting different ecological and evolutionary model components changes biodiversity patterns (34). In addition, the parameters of simulation models could be tailored to specific clades based on biogeographic reconstructions from molecular and fossil data to understand how processes such as long-distance dispersal have shaped present-day phylogenetic diversity.

## Methods

### Biodiversity Data Collection.

We obtained matching data on the geographic distribution and phylogenetic position of extant species of terrestrial vertebrates collected through the VertLife project (https://vertlife.org/) in association with Map of Life (https://mol.org/). Phylogenies were downloaded from VertLife and follow refs. 36 and 88–90. Distribution data for birds came from ref. 36, and for squamates, they came from the Global Assessment of Reptile Distributions (37). For mammals and amphibians, we modified distributions from the International Union for Conservation of Nature (IUCN) (38) to match the names of the respective phylogenies, and for squamate reptiles, we matched names following ref. 91. For plants, we used regional checklists of all 189 families presented in the Kew worldwide database (35). We used checklists corresponding to the most detailed “level3” polygons of botanical countries from the Taxonomic Databases Working Group (https://www.tdwg.org/). We investigated empirical patterns of diversity in 189 plant families and 78 vertebrate clades (bird, mammal, and amphibian orders and squamate reptile infraorders). We identified the subsets of these clades that were pantropically distributed, here defined as having at least one-third of the species found in the tropical moist forest biome (3) and occurring in all three of the considered biogeographic regions (Neotropical, Afrotropical, and Indomalayan) for vertebrates and botanical countries that overlap these biomes for plants. We did not consider species found in Madagascar in the Afrotropics. We quantified the evenness of the distribution of species diversity between the regions for the pantropically distributed empirical data (Fig. 1 *A* and *B* and Dataset S1). Distribution data for plants were less complete than the corresponding data for vertebrates, and so, we focused our quantitative analysis on vertebrate clades.

### Species Diversity and Contemporary Climate.

To characterize the relationships between present-day species richness and climate in each tropical region, we estimated vertebrate species richness in 110- × 110-km equal-area grid cells for birds, mammals, amphibians, and squamate reptiles separately across sites within the tropical moist forests on each continent. We also collated data on three major axes of environmental variation across tropical sites: MAT, MAP, and annual PET. MAT and MAP data were obtained from Chelsa at 30–arc s resolution (92), and PET data were obtained from Environmental Rasters for Ecological Modeling (ENVIREM) at 2.5–arc min resolution (93). All three variables were resampled at 110- × 110-km resolution using a Behrmann equal area projection to match the species distribution data (Dataset S2). We fitted GLS models of log (+1)-transformed species richness values, with MAT, MAP, and PET standardized to unit variance for the comparison of regression coefficients across variables measured in different units. To account for spatial autocorrelation in the model, we included a Gaussian correlation structure in the error term’s variance/covariance matrix. We fitted separate models for each vertebrate class and each continent, totaling 36 models.

### Simulation Model.

Paleoenvironment was reconstructed for the entire globe for the last 110 My at a temporal resolution of ∼170 ky and a spatial resolution of 2° and was characterized by approximate air surface temperature (related to MAT) and an aridity index (related to MAP and PET), following ref. 41 (Dataset S3). Air surface temperature was reconstructed by combining 1) paleotopography, estimated from paleoelevation models (94), with 2) reconstructions of paleo-Köppen climatic zones based on the geographic distribution of lithologic indicators of climate (95, 96), modified using the current temperature-lapse rate for each Köppen zone based on the current elevation and MAT downloaded from WorldClim2 (97). The aridity index was reconstructed from the paleo-Köppen bands and given a value of one for the arid Köppen regions and zero for all the other bands. We additionally modified the input in five ways for use in the landscape modification experiment. We reduced the temperature heterogeneity associated with orogenesis of the Andes region from 110 Ma, we held temperatures of the Indomalayan region at a constant value, we removed the cost associated with crossing water in the Southeast Asian archipelago, and we changed arid cells in the Afrotropics to nonarid cells from 110 and 23 Ma (further details on the reconstruction are in *SI Appendix*, Fig. S11).

We implemented the spatial model of diversification using the general engine for ecoevolutionary simulations, gen3sis (34). Each simulation followed the diversification of a clade from a single ancestral species distributed broadly throughout nonarid sites within 25° of the equator 110 Ma (Movie S1). We also tested the sensitivity of the pantropical diversity patterns to different initial ancestral ranges, including an exclusively extratropical ancestor, and found that the Afrotropics had lower diversity under these alternative starting conditions (further details are in *SI Appendix*, Fig. S12). Each simulation follows a clade’s radiation from the initial species throughout 110 My of reconstructed paleoenvironmental changes across 2° grid sites on the global landscape, considering four major processes: dispersal, environmental filtering, niche evolution, and speciation.

#### Dispersal.

At each time step (∼170 ky, 660 time steps in total), each population could disperse into surrounding grid sites from a dispersal kernel drawn from a Weibull distribution centered on 2° (∼222 km of latitude at the equator) with shape ϕ.

#### Environmental filtering.

The presence of species *i* in site *s* was determined by a match between the species temperature niche width ($\omega_{i}$) and the local temperature value $T_{s}$. Each species could be present in a site if $|T_{i}\pm \omega |>T_{s}$, where $T_{i}$ is the temperature niche center. One of the major constraints for the distribution of tropical moist forest taxa is water availability (40). In this study, the paleoaridity data are derived from a binary layer, thus limiting modeling in an aridity niche in a way comparable with that of the temperature niche. Therefore, to implement environmental filtering based on water availability, we place a hard constraint on species entering arid grid cells. This constraint prevents species from entering arid grid cells, and the assumption is supported by empirical evidence that biome shifts between forest and arid biomes are exceptionally rare (98). Extinction occurred when a species no longer occupied any grid cells as a result of mismatches between the species environmental niche and the environment.

#### Niche evolution.

Evolution of the temperature niche trait *T*_*i*_ followed a Brownian motion model of trait evolution, where the value of *T*_*i*_ at increasing time intervals of Δt is equal to the value of *T*_*i*_ at time *t*, plus a value drawn from a normal distribution with a mean of zero and SD of σ.

#### Speciation.

Speciation followed the biological species concept (99) in which species are considered reproductively isolated populations. Populations of a species that became geographically isolated from each other diverged genetically at each time step, and after divergence had crossed a speciation threshold (*S*), the populations became new distinct species. This equates to a Bateson–Dobzhansky–Muller model of genetic incompatibility (99).

We ran 500 simulations over a variable range of the four main model parameters, determined where possible based on empirical data and subsequently based on a preliminary exploration of the parameter space (*SI Appendix*): ω = [0.04, 0.1], corresponding to a niche width of ∼2.6 C to 7°C; ϕ = [2, 15], corresponding to a dispersal kernel with a right skew to include more long-distance dispersal values for low values of ϕ or a dispersal kernel with values centered more closely at 2° for higher values of ϕ; σ = [0.001, 0.02], corresponding to a range of the temperature variance of the Brownian function from 1.6 C to 1.3°C for each 170-ky time step, which is within the range found by ref. 100; and S = [1.5, 3], corresponding to a time interval of ∼2.5 to ∼6 My, which is based on estimated times for reproductive isolation to establishment (101). This range is towards the upper limit of the empirically estimated timing for speciation (102), however this was due to the constraints of computational feasibility—when the parameter value was low, reflecting very short speciation times, the number of species increases drastically, preventing the completion of the simulations (*SI Appendix*, Fig. S13). Due to the computational cost of running simulations, we sampled model parameters using Sobol sequences (Dataset S4), a quasirandom number generator that samples parameters evenly across the parameter space. Further details on the simulation model framework, model parameters, initial conditions, paleoenvironmental reconstructions, and landscape modification experiment are in *SI Appendix*.

In the complete simulations, we estimated species diversity within the tropical moist forests boundaries in the same way as for empirical data (Dataset S4). We fitted generalized linear models of the pantropical index (a binary variable representing whether simulations generated species in all three regions or not), the pantropical disparity index (a binary variable representing whether simulations generated the lowest diversity in the Afrotropics), and simulation parameters. We also aggregated simulated species richness to a 110- × 110-km resolution and a botanical country resolution to match the empirical vertebrate and plant distribution data, respectively. Then, as a measure of goodness of fit of the simulations, we estimated pairwise Spearman correlation coefficients of species richness across grid cells and botanical countries for each simulation with each pantropically distributed vertebrate and plant clade, respectively.

To investigate causation of uneven pantropical biodiversity in the simulation model, we manipulated paleoenvironmental reconstructions, subtracting key Earth history events to compare with unmodified simulations, following ref. 33. We performed key experimental manipulations focused on each tropical moist forest region addressing key hypotheses for the generation or suppression of biodiversity. In the Afrotropics, we removed the aridity constraint from either the Early Cretaceous or the Early Miocene. In the Neotropics, we removed the formation of the Andes. In Indomalaya, we reduced the dispersal distances between islands in the Southeast Asian archipelago and also removed the environmental heterogeneity associated with orogeny. We ran the simulation model across these five modified inputs using the parameters from the 10 best-fitting models (models that had the largest number of strong positive correlations with empirical clades showing a PDD pattern; Spearman’s ρ > 0.7). We ran each model three times to account for stochasticity. Further details on the experimental procedure and results can be found in *SI Appendix*.

### Macroevolutionary Analysis.

To identify trends in the spatial and temporal patterns of diversification, we estimated speciation and extinction rates in 1-My time slices for each of the 169 simulations that generated the PDD. Speciation and extinction rates were highly variable during the first ∼30 My of the simulation, owing to the stochasticity associated with the small number of species. Therefore, we considered the period from 110 to 80 Ma as a burn-in period, and we compared the distribution of these macroevolutionary rates from 80 My to present day between the Neotropics, Afrotropics, and Indomalaya. We used pairwise Wilcoxon signed-rank tests to test for a difference in the mean extinction and speciation rates across regions. We also investigated speciation and extinction rates separately for each geological era (Late Cretaceous, Paleocene, Eocene, Oligocene, Miocene, Pliocene, and Pleistocene) (*SI Appendix*, Fig. S6). To investigate how the evolution of the temperature niche–trait drove patterns of speciation and extinction across lineages, we looked at the evolution of this trait through time. We took the mean $T_{i}$ across all populations of each lineage at each time step and investigated how the distribution of the temperature–niche trait varied through different geological eras in a single simulation, matching Fig. 1*C* (*SI Appendix*, Figs. S8 and S9, and Movie S2).

To look at how different environmental features differed between tropical moist forest through time, we estimated which grid cells from the paleoenvironmental reconstruction corresponded approximately to this biome across different biogeographic regions through time by defining these cells as those with MAT > 18°C and aridity index = 0. This equates to a rough approximation of a megathermal tropical environment likely to be dominated by tropical moist forests (15) (Movie S3). We then recorded changes in mean temperature (degrees Celsius), temperature variance (SD; degrees Celsius), mesic area (kilometers^2^), and habitat fragmentation (*SI Appendix*, Figs. S4 and S7). Habitat fragmentation was estimated as the proportion of disconnected sites relative to area over time (*SI Appendix*, Fig. S4). A fragmentation value of 100 would mean that each cell in a tropical region is a unique cluster, while values close to zero mean that all cells in the region are connected as a single cluster during the period.

### Phylogenetic Assemblage Structure.

To understand the relative role of dispersal compared with in situ diversification dynamics in structuring the phylogenetic relatedness of species within tropical moist forests, we calculated the NRI, a measure of the phylogenetic distance between co-occurring species in an assemblage standardized by the expected phylogenetic distance under a null model of community assembly (84). We used the independent swap null model, which maintains richness of sites and frequencies of species in the dataset, using the mpd.ses function in the picante package in R (103). We estimated NRI across biomes in different regions, as biomes represent evolutionary arenas of diversification suitable for comparison (3). We calculated the NRI for all 23 vertebrate clades showing a PDD pattern using a randomly sampled phylogeny from the posterior distribution of the respective taxon (Dataset S5). We also calculated the NRI for palms across botanical countries, possible due to the well-sampled phylogeny available for this clade, which includes placing species based on morphological and taxonomic data where molecular data were unavailable (104). As done with the comparison of species richness, we calculated pairwise Spearman correlation coefficients of NRI between empirical and simulated datasets.

## Supplementary Material

Supplementary File

Supplementary File

Supplementary File

Supplementary File

Supplementary File

Supplementary File

Supplementary File

Supplementary File

Supplementary File

## Data Availability

Data and R scripts used in this study are deposited in a public repository on EnviDat, https://www.envidat.ch/dataset/data-from-hagen-skeels-etal-pnas. All other data are included in the manuscript and/or supporting information. Previously published data were also used for this work: vertebrate phylogenetic data (36, 88–90), vertebrate distribution data (37, 38), plant distribution data (35), and palm phylogenetic data (105).
