# Supplementary material for: Earth history events shaped the evolution of uneven biodiversity across tropical moist forests
Source: Proc Natl Acad Sci U S A. 2021 Oct 1;118(40):e2026347118. doi: 10.1073/pnas.2026347118 (PMC8501849; doi:10.1073/pnas.2026347118)
Supplement: Supplementary File [file pnas.2026347118.sapp.pdf]

PNAS
www.pnas.org

Supplementary Information for

Earth history events shaped the evolution of uneven biodiversity across tropical forests

Oskar Hagen, Alexander Skeels, Renske E. Onstein, Walter Jetz and Loïc Pellissier

Corresponding Author: Oskar Hagen, Alexander Skeels.
E-mail: oskar@hagen.bio; alexander.skeels@gmail.com

This PDF file includes:

Supplementary text
Figs. S1 to S13
Legends for Movies S1 to S3
Legends for Dataset S1 to S5
SI References

Other supplementary materials for this manuscript include the following:

Movies S1 to S3
Datasets S1 to S5

Supporting Information Text

A. Paleoenvironmental data and reconstructions.

We reconstructed gridded paleo-temperature and paleo-aridity across the entire globe at 2° resolution from 110 Ma. Following (1), the approximate air surface temperature and aridity index for the entire globe was calculated from available paleoelevation models and lithologic indicators of climate. Plate tectonic and paleogeographic digital elevation models providing paleotopography at a 1° resolution were obtained from Scotese's Paleoatlas of the Earth (2). Paleotopographies were estimated by combining information on the dynamics of sea floor spreading, continental rifting, subduction, continental collisions and other isostatic events on plate tectonic reconstructions, together with other indicators of paleotopography and bathymetry (2). We further used reconstructions of Köppen climatic zones plotted on paleoreconstructions at intervals of every five Myr(3). The basic Köppen classification depends on average monthly values of temperature and precipitation and has five primary climatic zones: tropical ever wet, subtropical arid, warm temperate, cold temperate and polar. Reconstructions of the ancient Köppen zones are based on the geographic distribution of lithologic indicators of climate, including coal, evaporite, bauxite, tillite, glendonite, dropstones and other fossil evidence, such as high-latitude occurrences of palm, mangroves and alligators (3–5). A complete description of the sources of these lithologic indicators of climate can be found in (4). The five principal Köppen climatic zones were drawn over twelve Cenozoic paleotopographic reconstructions according to the distribution of these lithologic indicators of climate. The average temperature of each of the modern Köppen zones was then calculated on the basis of present global temperature estimations. Modern temperatures served as the initial estimate of the temperature of each of the Köppen zones, which were then adjusted in order to match global mean temperature change over the Cenozoic (6). The Köppen zones provide an estimate of the average surface temperature but do not account for topographic features. To account for the decrease in temperature with elevation, we computed the current temperature lapse rate (i.e. the rate of decrease in temperature with elevation) for each Köppen zone based on the current digital elevation model and the annual mean temperature raster of WorldClim2 (7) (see dataset S3 for reconstructed temperature values). Finally, the aridity index was set at one for regions defined as arid by the Köppen band reconstructions and zero for all the other bands. The reconstructions provided two primary layers for the gene3sis simulations; the aridity index [0,1] and temperature [-37.621, 27.983]. Topography was expressed indirectly through the temperature layer, and high elevation regions could present barriers to dispersal by presenting climatic barriers. Landscape costs for dispersal were given as 1 for land cells and 2 for water cells.

B. Landscape modifications.

We additionally modified the reconstructed landscapes in five ways in order to test the local effects of landscape features (Fig. S11). First, we reduced the elevation of the Andes region from 110 Ma to a constant elevation of 0 m above sea level to measure the effects of mountain building in the Neotropics. Second, we held temperatures in the Indomalayan region at a constant value from 110 Ma using the mean temperature value within a defined polygon in order to measure the effects of temperature heterogeneity associated with orogeny in Indomalaya. Third, we set the cost associated with crossing water to zero in the Southeast Asian Archipelago from 110 Ma, thus connecting all islands in the archipelago of the Indomalayan region in order to test for the effects of island isolation dynamics in the region. Fourth and fifth, we changed arid cells in the Afrotropics to non-arid cells from 110 Ma and from 23 Ma in order to measure the effects of aridity in the Afrotropics from the Early-Cretaceous and Early-Miocene until the present.

C. Paleo-habitat fragmentation analysis.

The approximate total area in km^2 for each tropical region (MAT $> 18°$C and aridity index $= 0$) was measured over time. The number of fragments in each tropical region was calculated based on the least-cost geographical distance between points using the functions *transition* and *costDistance* in the R package gdistance (8) and clustered using the density-based spatial clustering implemented in gen3sis (9). Similarly to a DBSCAN with eps=1 and minPts=1, we considered all non-tropical regions (MAT $< 18°$C or mean aridity index $= 1$) uncrossable and considered all eight adjacent sites connected. The relative proportion of fragments was the total number of clusters divided by the regional total area (Fig. S4).

D. Gen3sis model description.

We describe the simulation model according to the Overview, Design concepts, Details (ODD) protocol for detailing agent-based models of Grimm et al. (10).

1. Overview

1.1. Purpose

We implemented the spatial model of diversification using the general engine for eco-evolutionary simulations, *gen3sis* (9). The gen3sis engine simulates the evolution of lineages across a dynamic landscape and allows users to modify the specific evolutionary and ecological mechanisms that shape dispersal, trait evolution, speciation, and extinction, all in interaction with user-defined environmental layers over deep time. The model generates patterns of species' distributions, species traits and phylogeny by tracking the distribution of species at each time-step and recording the relationships between species and all speciation and extinction events. This simulated data is comparable to empirical biodiversity data and can be used to compare the model output to empirical biodiversity patterns. The gen3sis framework can be used to understand which combinations of mechanisms and parameters under a dynamic landscape generate biodiversity patterns consistent with empirical data. In this

study we compared the simulated distribution of species diversity and phylogenetic clustering (net relatedness index; NRI) between three major tropical moist forest regions to investigate the origin of uneven biodiversity across this biome.

1.2. Variables and scales

The model links the gridded multi-dimensional geographic space of the input landscape to the diversification and range evolution of species based on the match between the environmental values of the landscape and the environmental temperature and aridity niche of species. Each grid cell contains values of temperature and aridity based on paleo-reconstructions (above), with values given as NA for water (e.g. oceanic cells). The input data consists of 16,200 grid cells at a resolution of 2° and global extent of -180° to 180° longitude and -90° to 90° latitude. The fundamental unit of the simulation is a population, here defined as the population of a species in an single grid cell. Each population has a geographic location (latitude and longitude), as well as a temperature position/centre (T_i) and a temperature niche width (ω), which together define the range of temperature values the population can occur in. Populations that are geographically connected form clusters (meta-populations) and all geographic clusters together comprise a species. Dispersal and ecological interactions occur at the level of the population, speciation occurs between diverging meta-populations, and here we track evolution of the environmental niche and the phylogeny at the level of species. The simulation begins at 110 Ma and progresses to present day in discrete time-steps of 170 kyr, totalling 660 time-steps altogether.

1.3. Process Overview and Scheduling

At each interaction cycle the following operations occur: (1) The input landscape changes according to the paleo-reconstruction used. (2) Populations disperse into surrounding grid cells based on distances drawn from the dispersal kernel. (3) Presence or absence of each population in each grid cell is determined by matching the population's temperature niche to the value of the grid cell – here populations and/or species may become extinct. (4) Geographic clusters of a species accumulate genetic divergence, and clusters that have diverged past a threshold (S) become new species. 5) A species' environmental niche evolves following a Brownian motion model. The simulation is finished when the final time-step is reached or based on the following conditions: (1) All species in the simulation are extinct. (2) The number of species in the simulation exceeds 12,500 (computationally unfeasible; see below). (3) The number of species in a grid cell exceeds 7500. The simulation is repeated with different values of the four variable parameters: the rate of temperature niche evolution (σ) measured in standard deviations of a normal distribution from which temperature deviations are drawn; the temperature niche width (ω), the shape parameter of the Weibull distribution that determines the dispersal kernel (ϕ), and the amount of divergence required for speciation, or in other words, the time for speciation completion in diverging populations (S). Combinations of parameter values are determined by sampling with Sobol sequences between determined ranges (Table S1), which are used to sample the multi-dimensional parameter space evenly and efficiently (11).

Table S1. parameter range

Parameter	minimum	maximum
S	1.500	3.000
σ	0.001	0.020
ω	0.040	0.100
ϕ	2.000	15.000

2. Design Concepts

Emergence: The model simulates the distribution of species, the evolution of their traits and the genealogy of relatedness. From these factors many patterns emerge, including species richness, phylogenetic and function diversity, and phylogenetic tree shapes. In this study we focused on two emergent patterns – species richness and phylogenetic diversity. We looked at how these patterns are distributed longitudinally, between the tropical regions, and latitudinally.

Adaptation: The environmental niche position of species evolves following Brownian motion. Species that have poorly adapted niche traits in the grid cells they occupy will go extinct locally, and possibly go extinct completely if the species is not well adapted across it's complete range. The environment therefore will select for well adapted species and guide the evolutionary trajectories of lineages.

Fitness: Fitness is measured as a binary trait. Species are either adapted or maladapted to the environment they are found in. This will determine presence or absence in the grid cell. *Prediction:* Evolution is undirected and follows a random walk under a Brownian Motion model.

Sensing: Species presence in grid cells is determined by three factors which guide their dispersal across the landscape – temperature, aridity and presence of water bodies. Species can occur in cells in which temperature is suitable. They cannot occur in arid cells or in water.

Interaction: Although the gen3sis framework can account for inter-specific interactions, no inter-specific interactions were modelled in this study.
Stochasticity: Stochasticity features in several aspects of the model. Niche evolution is stochastic, with changes in a species niche at each time-step being drawn from a normal distribution with a standard deviation of σ. Dispersal is stochastic, as the dispersal distance for each population is drawn from a Weibull distribution with scale = 222 and shape = ϕ.

Collectives: The basic unit is the population in a grid cell. These form clusters of populations that are geographically connected. Species are comprised of all geographic clusters which have not yet diverged to form new species.

Observation: The data is summarized into patterns of species richness by stacking the distribution of each species in each grid cell in the model. This is done at each time-step. We also define which grid cells are tropical throughout the simulations based on a crude threshold. We then look at the emergence and extinction of species in each region through time and calculate spatially-explicit measures of speciation and extinction. Finally, we look at patterns of phylogenetic clustering by matching species distributions to phylogeny.

3. Details

We ran gen3sis v1.1 in R v3.6.3 using a high-performance computing (HPC) cluster.

3.1. Initialization

The model begins with a single species placed in all non-arid equatorial grid cells (between -23.5°S and 23.5°N) at 110 Ma. Each population of the species is given the temperature niche value of the grid cell it is found in. The simulation follows the dispersal, evolution and diversification of this ancestral species across the globe. The placement of the ancestral species follows the simplifying assumption that the ancestor of contemporary pantropical lineages was also pantropically distributed, rather than that the pantropical distribution was achieved later via long distance dispersal. In some cases this is not the case; however, the initial conditions need to be made general when comparing different taxa of different evolutionary ages. We describe the sensitivity of the results to the initial conditions below.

3.2. Input

We modelled the diversification of lineages across 110 myr of temperature and aridity changes at a global scale. Topographic events, such as orogeny, are implicit in the temperature values of the landscape and we did not model an additional altitudinal variable. See above for more information on the generation of input data.

3.3. Submodels

- Speciation For each species at each time-step, the speciation function performs the following operations. (1) Identify the geographic range of all populations comprising a species. (2) Cluster populations that are geographically contiguous based on the dispersal capacity of the species and the landscape costs (see dispersal below). (3) Add a divergence factor to geographically isolated populations. A divergence factor of 0.1 is added to each isolated population at each time-step, so divergence occurs linearly with time-since-divergence. (4) Subtract a divergence factor from re-united geographic clusters (those that have come in secondary contact after an initial period of isolation). (5) Determine when populations have crossed a threshold of divergence (S) after which they are treated as new species and evolve independently.
- Dispersal: At each time-step, each population can disperse into the surrounding grid cells. The distance over which the species can disperse is drawn from a Weibull distribution centred on 2° (one grid cell and approximately 222 km of latitude at the equator) with shape ϕ. We model the cost of dispersing over water cells as double the cost of dispersing over land cells.
- Ecology: The presence of a population in a grid cell is determined by a match between the range of temperature conditions the species can tolerate (species temperature optima ± species temperature niche width) and the local temperature value T_s. Each species can be present in a site if $|T_i \pm \omega| > T_s$, where T_s is the temperature value at site s, T_i is the temperature niche position and ω is the temperature niche width, for population i. Additionally, species are constrained to be unable to occupy cells that are arid. Local extinction occurs when a grid cell does not fall within a population's niche width from the optimum. Species-level extinction occurs when a species no longer occupies any grid cells as a result of mismatches between the species environmental niche and the environment.
- Niche evolution: Evolution of the temperature niche trait T_i for each species follows a Brownian motion model of trait evolution, where the value of T_i at increasing time intervals of Δt (170 kyr) is equal to the value of T_i at time t, plus a value drawn from a normal distribution with a mean of zero and standard deviation of σ.

4. Simulations

We ran 500 simulations covering a range of parameters in order to capture a range of possible biodiversity outcomes based on the parameters and constraints in the model. The number of simulations is heavily restricted by computational power. Working within the constraints that computational feasibility present, we used Sobol sequences as a way of reducing the number of simulations required to evenly sample parameter space to get an unbiased coverage of parameter combinations.

5. Landscape modification simulation experiment

We ran the simulation model using five modified landscapes as input (described above) and repeated this process across 10 parameter combinations that provided the best fit to empirical data (Table S2). The best fitting models were determined by ranking the simulations by the number of positive Spearman correlation coefficients > 0.7 for correlations of species richness across grid cells between empirical clades showing the pantropical diversity disparity pattern (PDD). The simulation models that had the strongest correlations were deemed the best candidates for showing the evolution of the PDD and provided a baseline to see the effect of the landscape modifications on the PDD. We selected the parameters from these 10 best-fitting models and ran these each three times to account for stochasticity for each of the 5 modified landscapes, totalling 150 additional simulations. To quantify the effect of the landscape modification, we quantified whether the distribution of diversity between the original simulations and the modified landscape simulations in the region of interest (e.g. the Andes modification was focused on the Neotropics) differed significantly using paired Wilcoxon signed-rank tests.

Table S2 Parameters of the best fitting gen3sis models

Model	σ	ω	S	ϕ
2D_FA_104	0.005	0.07	2.62	13.90
2D_FA_124	0.006	0.06	2.66	10.23
2D_FA_231	0.004	0.09	2.67	5.80
2D_FA_232	0.005	0.05	2.20	3.35
2D_FA_280	0.004	0.06	1.78	9.34
2D_FA_344	0.005	0.05	2.05	6.79
2D_FA_375	0.005	0.07	2.16	9.85
2D_FA_431	0.003	0.08	1.89	8.37
2D_FA_440	0.004	0.06	2.22	14.49
2D_FA_472	0.004	0.06	2.37	10.92

Our results show that in simulations with aridity constraints removed in the Afrotropics from the Miocene onwards, Afrotropical tropical moist forests were not significantly more diverse than the unmodified simulations (Wilcoxon signed-rank test, P=0.07; Fig. S6), and in some cases were marginally less diverse. However, Afrotropical tropical moist forests in simulations with the aridity constraint removed from the beginning of the simulation, 110 Ma, were significantly more diverse than unmodified simulations (P=0.027) and more diverse than those with aridity removed from the Miocene (P=0.01172; Fig. S6), reversing the PDD in approximately 66% of simulations. We also found significant differences in the distribution of Neotropical diversity compared with unmodified simulations (paired Wilcoxon signed-rank test, P<0.001; Fig. S6), with drastic declines in diversity in modified input, reversing the PDD in more than 90% of modified simulations. To investigate the origins of Indomalayan biodiversity and tease apart the effects of island isolation and mountain building, we removed the cost of dispersal over water, as well as the environmental heterogeneity present from orogenesis. We found that the Indomalayan tropics were not significantly less diverse in modified landscapes when the effect of island isolation was removed (P=0.92; Fig. S6). However, they were significantly less diverse when heterogeneity associated with mountain uplift was removed (P=0.002; Fig. S6), reversing the PDD in >80% of the modified simulations.

6. Initial conditions simulation experiment

The initial placement of the ancestral lineage at the beginning of each simulation may influence biodiversity outcomes. We set the initial species to occupy a range covering the equatorial mesic tropics: grid cells between -25°S and 25°N which had aridity values = 0. This decision was taken to remove the bias of having species colonise different biogeographic regions at different times, thereby removing a time-for-speciation effect. The timing of the placement of this ancestral species at 110 Ma corresponds with the separation of the African and American continents. As such, following the initial speciation events separating lineages on each continent, we could track the macroevolutionary dynamics of three independent radiations. However, the location of the ancestral species may bias biodiversity outcomes if species are absent from higher latitudes during the early phases of a radiation. For example, some clades have been proposed to have originated in Laurasia during the Mid-Late Cretaceous (e.g.(12)). To see the effect of a broader distribution of the ancestral lineages we also ran the simulation model including parts of Laurasia (-25°S and 40°N; Fig S12) in the ancestral distribution as well as a scenario in which the initial ancestral distribution was placed exclusively outside of the tropics (grid cells > -25°S and < 25°N; Fig S12).

Similarly to the landscape modification experiment, we ran the simulation using parameters from the 10 best-fitting models, and compared the distribution of specie richness between tropical moist forest regions to see the effect on pantropical diversity patterns. We found that in some circumstances increasing the ancestral distribution led to a geographic area for speciation to occur and the number of species exceeded the computational threshold of 12,500. To account for this we ran the 10 best-fitting simulation models again increasing the divergence threshold parameter (S) by 0.5 and again by 1.0 for a total of 30 simulations.

This increased the duration of speciation and promoted lower overall diversity, allowing the simulation models to complete and diversity patterns in the present-day to be compared. We found that in both initialisation modifications, a strong PDD emerged, with the Afrotropics having less diversity than the Neotropics and Indomalayan tropics (Fig S12). This suggests that the general PDD pattern is not strongly sensitive to the initialisation conditions. There were however, some differences in biodiversity patterns between the modified and original simulations. We found that diversity in the Indomalayan tropics was generally higher, while Neotropical diversity was proportionally less, particularly when the ancestral species was extra-tropical. Furthermore, the Afrotropics had drastically reduced diversity when the ancestral species was exclusively extra-tropical, with in many cases no species present in the the Afrotropics in the final time-step. This was driven by a wide band of aridity in Northern Africa acting as a dispersal barrier for colonisation from Eurasia, while dynamic and spatially fluctuating aridity drove extinctions of Southern African lineages.

7. Speciation duration simulation experiment

In the simulation model, speciation follows the biological species concept (13) in which species are considered completely reproductively isolated populations. Populations of a species that become geographically isolated from each other diverge genetically at each time-step, and once divergence has crossed a speciation threshold (S) the populations become new distinct species. This equates to a Bateson–Dobzhansky–Muller model of genetic incompatibility (13). The range of speciation times varied between approximately 2.5 Ma (15 time-steps) and 5 Ma (30 time-steps). Empirical estimates for the timing to achieve reproductive isolation vary and have been estimated to range from 1 to 4+ Myr in primates (14). However, this differs from estimated speciation times (15), which on average are $<$1 Myr.

We explored how reducing the duration of speciation effects species diversity, computational time and the PDD by running an experiment where we took the parameters of the single best-fitting model (see above) and systematically reduced the parameter value of S from 26 (4.4 Myr; the original parameter value) to 2 (340,000 yrs; the lower end of the speciation duration spectrum) by 1.3 (220,000 yrs) for a total of 20 simulations. We then determined the number of species, the computation time of the simulation, and the distribution of species in each TMF biome to see if patterns would differ substantially. The simulations ran until they (i) finished at time-step 0, or (ii) reached a threshold for the maximum number of species (12,500) and finished at time-step x. We found that the species generation rate (the ratio of the number of species at time-step x with the number of time-steps) increased exponentially with S (Fig. S2). When S was below 15 (approximately 2.5 Myr) species diversity quickly went above 12,500, which is larger than all vertebrate clades investigated and most plant clades, with the exception of several hyper-diverse groups including the Orchidaceae. Computation time also rapidly increased with decreasing values of S (Fig. S13). In all simulations Indomalaya had a greater tropical moist forest diversity than the Afrotropics, whereas the Afrotropics had a greater diversity than the Neotropics in only two simulations, and in one case the difference was only marginal (Fig. S13). This suggests that while decreasing S exponentially increases species diversity and computation time, it does not drastically effect the presence of a PDD, with the Afrotropics still having fewer species than Indomalaya and the Neotropics in most cases.

Table S3 Generalized Least Squares (GLS) regression results of species richness in four terrestrial vertebrate clades (birds, mammals, amphibians and squamate reptiles) and with three environmental predictors (mean annual temperature [MAT], mean annual precipitation [MAP], and potential evapotranspiration [PET]). We present the GLS slope estimates (slope), standard error (se), p-value (P) and P-value corrected for multiple comparisons (Holms).

	slope	se	*P*	Holms	region	var	clade
1	0.02	0.01	0.04	1.00	Indomalaya	MAT	amphibians
2	-0.03	0.00	0.00	0.00	Neotropics	MAT	amphibians
3	-0.01	0.01	0.03	1.00	Afrotropics	MAT	amphibians
4	0.00	0.01	0.37	1.00	Indomalaya	MAT	mammals
5	0.00	0.00	0.70	1.00	Neotropics	MAT	mammals
6	-0.00	0.00	0.73	1.00	Afrotropics	MAT	mammals
7	0.00	0.00	0.39	1.00	Indomalaya	MAT	birds
8	-0.01	0.00	0.01	0.39	Neotropics	MAT	birds
9	-0.00	0.00	0.47	1.00	Afrotropics	MAT	birds
10	0.01	0.03	0.64	1.00	Indomalaya	MAT	squamates
11	0.04	0.01	0.02	0.73	Neotropics	MAT	squamates
12	-0.04	0.02	0.03	1.00	Afrotropics	MAT	squamates
13	0.00	0.01	0.85	1.00	Indomalaya	MAP	amphibians
14	0.01	0.00	0.24	1.00	Neotropics	MAP	amphibians
15	0.01	0.01	0.16	1.00	Afrotropics	MAP	amphibians
16	0.01	0.00	0.07	1.00	Indomalaya	MAP	mammals
17	0.00	0.00	0.30	1.00	Neotropics	MAP	mammals
18	-0.00	0.00	0.78	1.00	Afrotropics	MAP	mammals
19	0.00	0.00	0.52	1.00	Indomalaya	MAP	birds
20	0.00	0.00	0.26	1.00	Neotropics	MAP	birds
21	-0.00	0.00	0.39	1.00	Afrotropics	MAP	birds
22	0.04	0.02	0.15	1.00	Indomalaya	MAP	squamates
23	0.07	0.01	0.00	0.00	Neotropics	MAP	squamates
24	0.03	0.02	0.18	1.00	Afrotropics	MAP	squamates
25	0.09	0.03	0.01	0.48	Indomalaya	PET	squamates
26	0.06	0.01	0.00	0.00	Neotropics	PET	squamates
27	-0.01	0.02	0.47	1.00	Afrotropics	PET	squamates
28	0.02	0.01	0.10	1.00	Indomalaya	PET	amphibians
29	-0.03	0.00	0.00	0.00	Neotropics	PET	amphibians
30	-0.01	0.01	0.31	1.00	Afrotropics	PET	amphibians
31	0.01	0.01	0.23	1.00	Indomalaya	PET	mammals
32	0.00	0.00	0.29	1.00	Neotropics	PET	mammals
33	-0.00	0.00	0.33	1.00	Afrotropics	PET	mammals
34	0.01	0.00	0.05	1.00	Indomalaya	PET	birds
35	-0.00	0.00	0.07	1.00	Neotropics	PET	birds
36	0.00	0.00	0.51	1.00	Afrotropics	PET	birds

Table S4 Generalized linear model of the pantropicaly index and four model parameters. The pantropical index is a binary variable recognizing whether a simulation generated diversity in all three of the tropical moist forest regions (Afrotropics, Indomalaya and Neotropics).

	Estimate	Std. Error	z value	Pr(>|z|)
(Intercept)	0.4871	0.1517	3.21	0.0013
σ	-2.4620	0.2344	-10.50	0.0000
ω	0.3266	0.1521	2.15	0.0317
S	-0.3871	0.1545	-2.51	0.0122
ϕ	0.0886	0.1482	0.60	0.5500

Table S5 Generalized linear model of the pantropical disparity index and four model parameters. The pantropical disparity index is a binary variable recognizing whether a simulation generated lower diversity in the Afrotropics than in Indomalaya or the Neotropics.

	Estimate	Std. Error	z value	Pr(>|z|)
(Intercept)	-0.4202	0.1254	-3.35	0.0008
σ	-1.4641	0.1544	-9.49	0.0000
ω	0.3180	0.1269	2.51	0.0122
S	-0.4453	0.1303	-3.42	0.0006
ϕ	0.0408	0.1232	0.33	0.7406

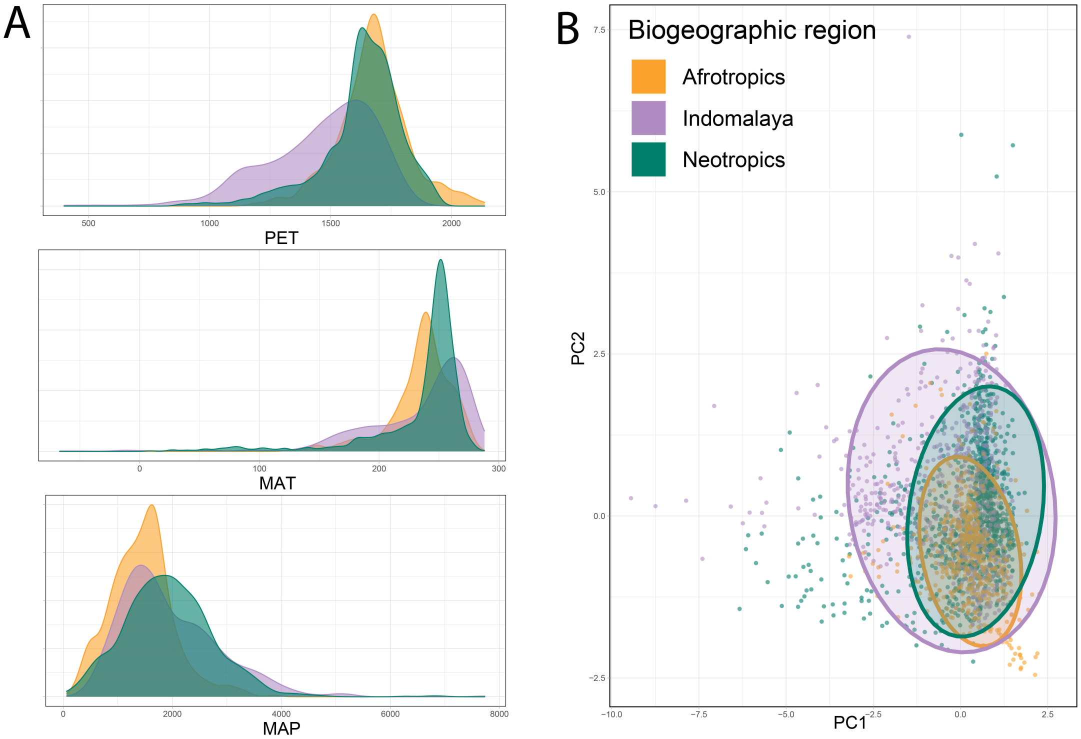

Fig. S1. Distribution (A) and PC1 and PC2 of a principal component analysis (B) of potential evapotranspiration (PET), mean annual temperature (MAT) and mean annual precipitation (MAP) across the tropical moist forest biome in three regions (Afrotropics, Indomalaya and Neotropics) at 110 km x 110 km resolution grid cells. Ellipses surround 95% of observed data in each region.

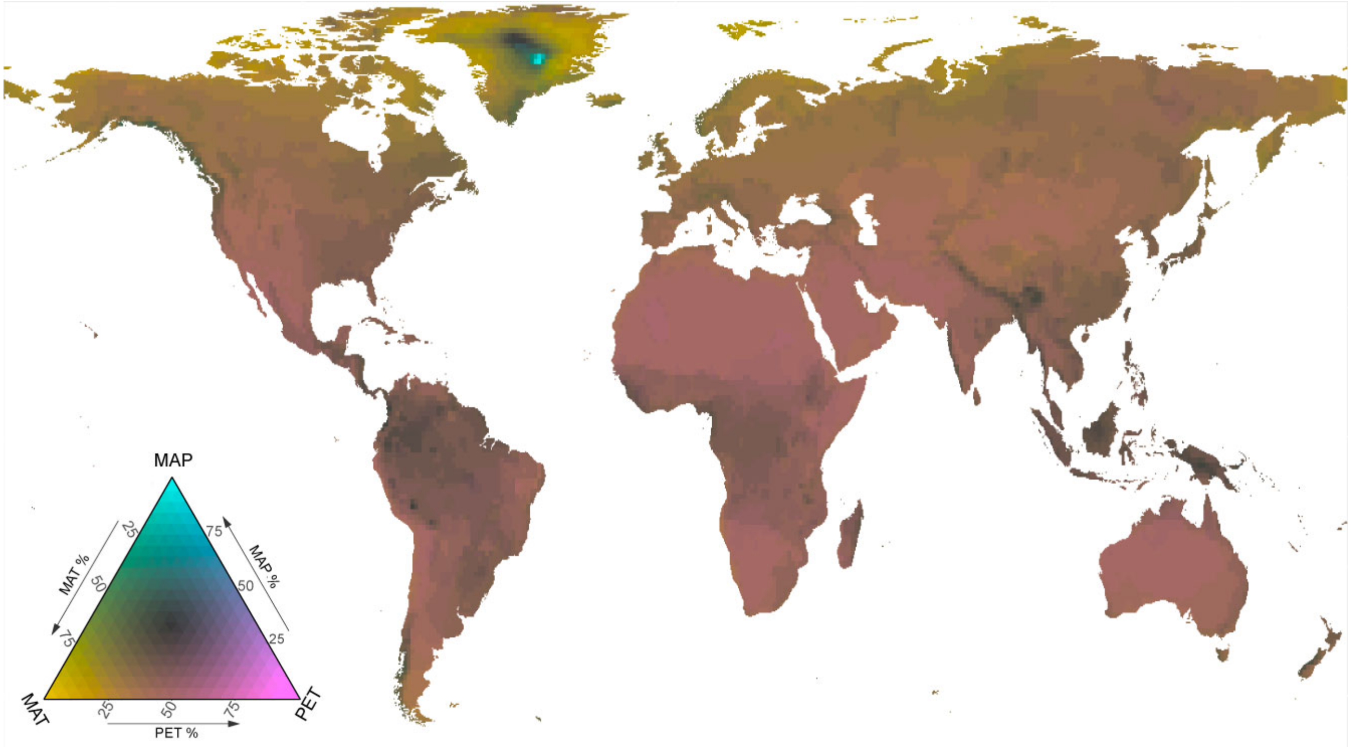

Fig. S2. Global ternary colour coding of three normalized key environmental variables: mean annual temperature MAT [-27.4, 31.4 °C], mean annual precipitation MAP [0, 7921 mm/year] and potential evapotranspiration PET [51.8, 2328 mm/year].

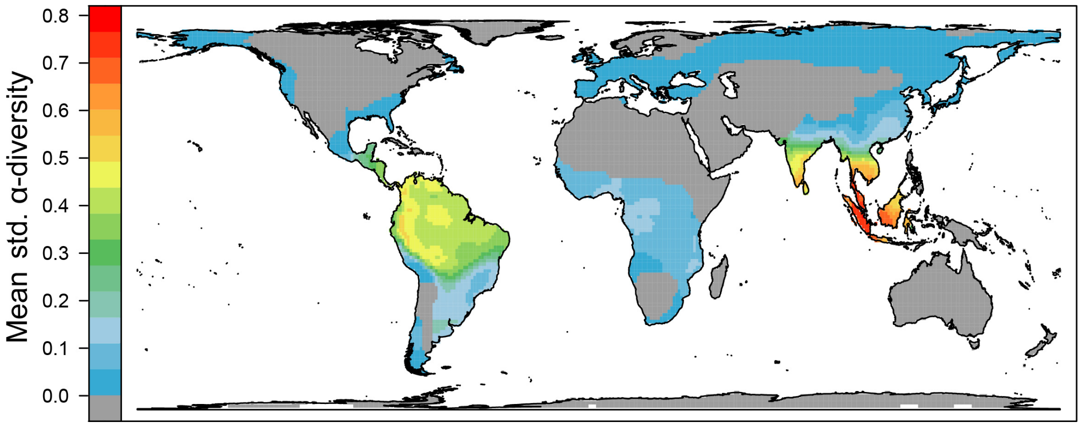

Fig. S3. Mean standardized species richness across simulations which generated uneven pantropical diversity, with lowest diversity present in the Afrotropics (n=169).

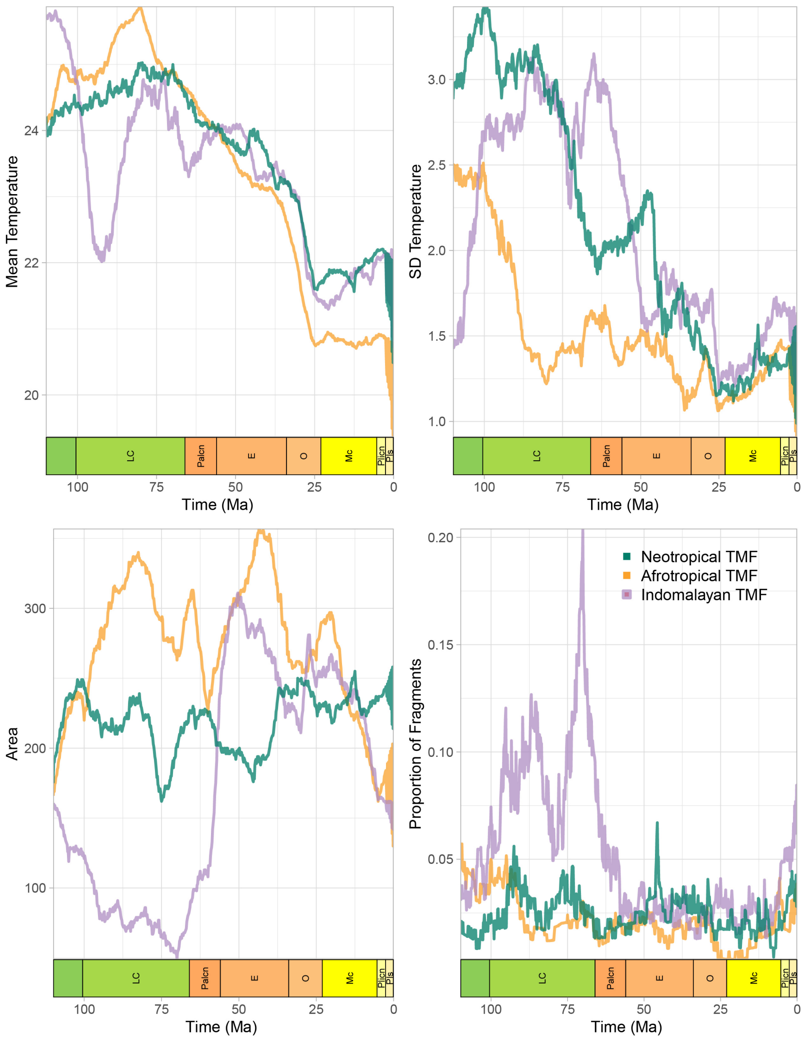

Fig. S4. Paleoenvironmental dynamics for each tropical region. Mean temperature (°C), standard deviation (SD) of temperature, approximate area (grid cells), and relative proportion of fragments (disconnected sites) are shown.

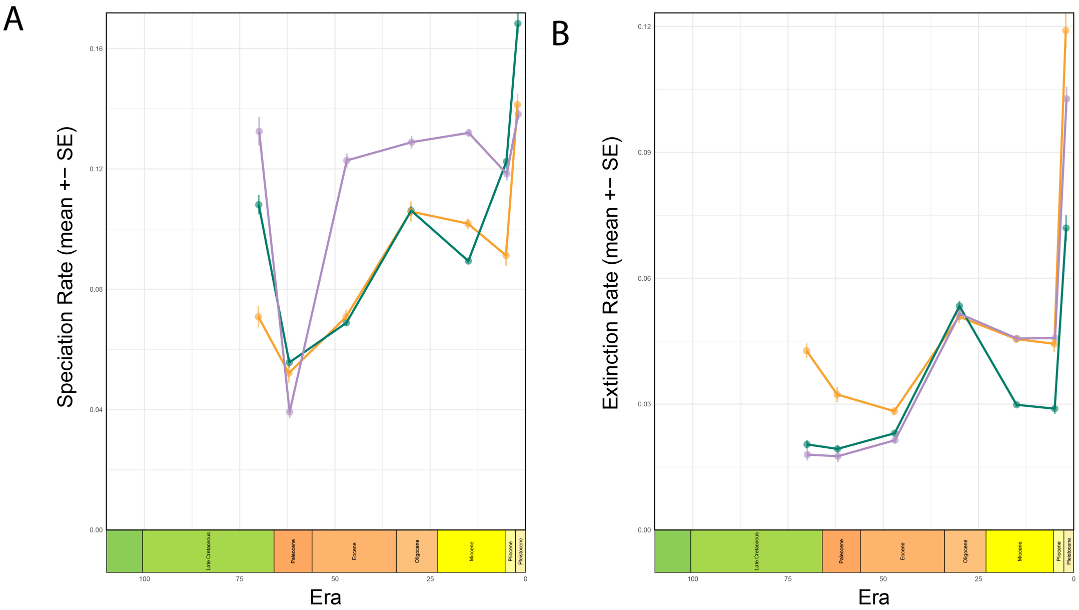

Fig. S5. Mean speciation rates $\pm SE$ (A) and mean extinction rates $\pm SE$ (B) across geological eras (Late Cretaceous, Paleocene, Eocene, Oligocene, Miocene, Pliocene, Pleistocene) for tropical moist forest regions in the Neotropics, Afrotropics and Indomalaya estimated from simulations which generated uneven pantropical diversity, with lowest diversity present in the Afrotropics (n=169).

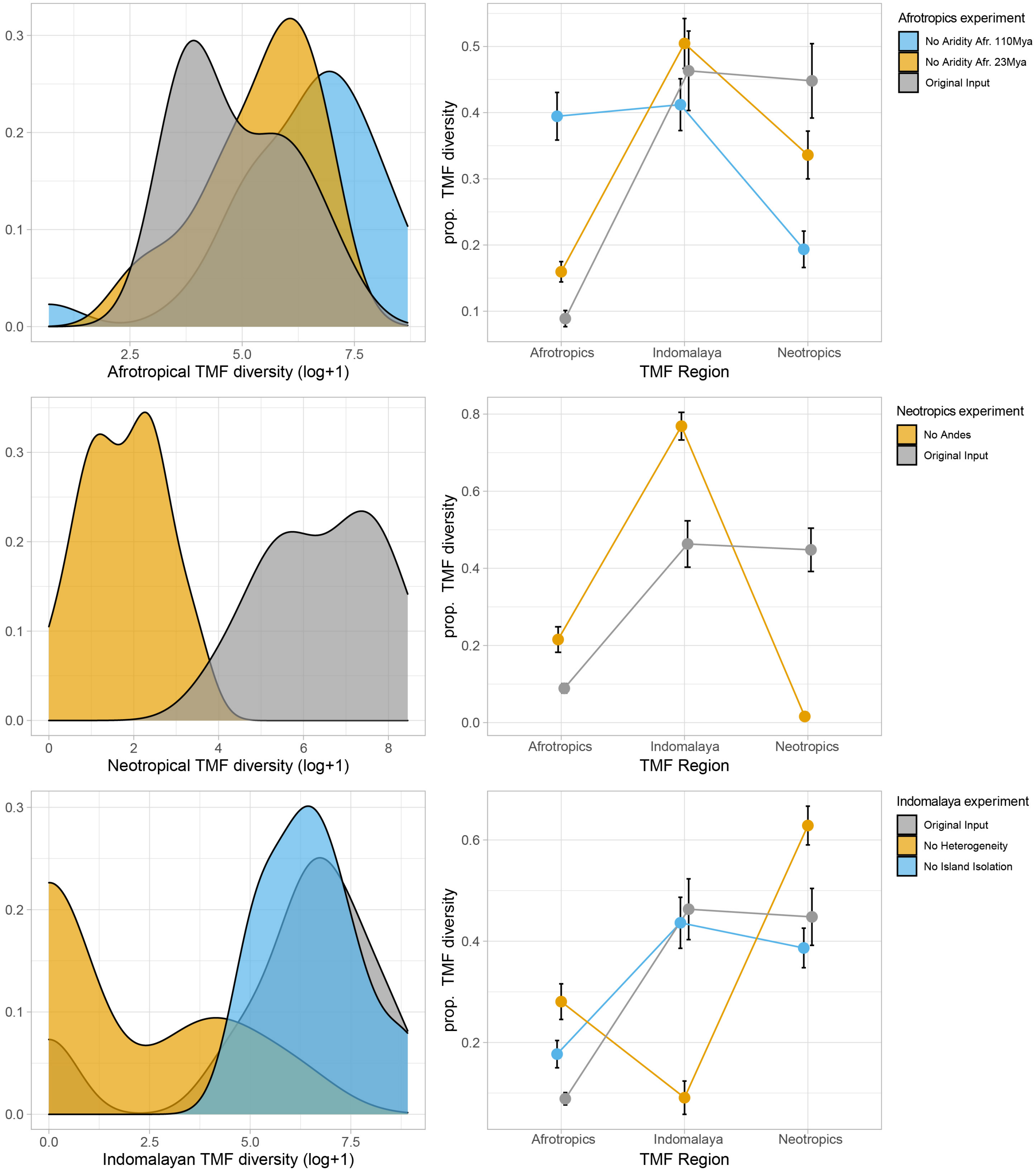

Fig. S6. Distribution of species diversity in three tropical moist forest (TMF) biogeographic regions between simulations run on the original reconstructed input landscape (grey) and those run on five different modified landscapes (yellow and blue). Density plots show species richness for the focal region of interest of each experimental manipulation (left column). Line plots show the mean proportion of species diversity ($\pm$ 95% confidence intervals) found in each of the three considered TMF regions for each experimental manipulation, highlighting disparity between regions.

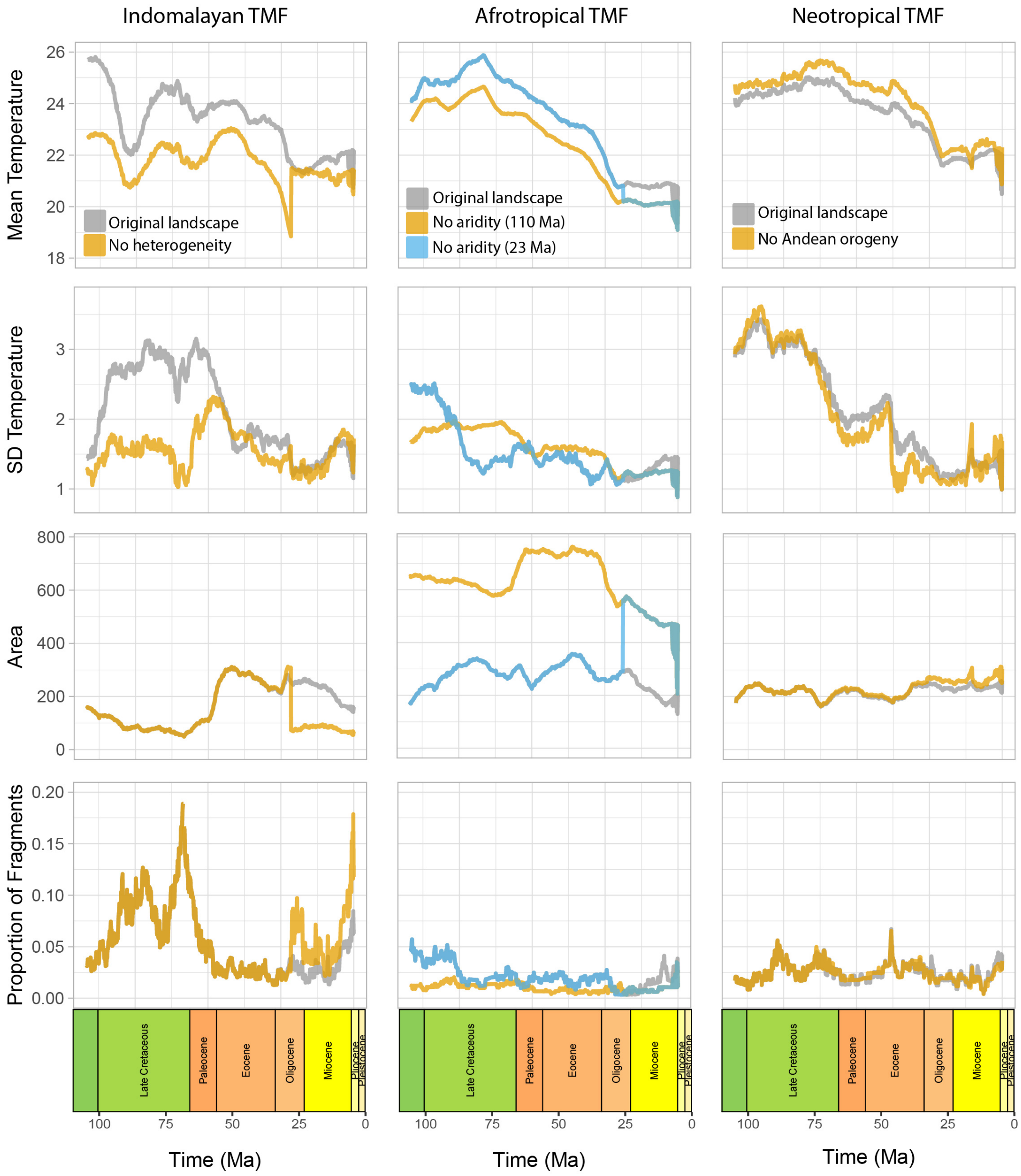

Fig. S7. Paleoenvironmental dynamics in each tropical moist forest (TMF) region between the original and modified input landscapes. Modified inputs involved removal of temperature heterogeneity associated with orogeny in Indomalaya (column 1), removal of the aridity constraint in the Afrotropics from 110 Ma and from 23 MA (column 2), and removal of temperature heterogeneity associated with Andean orogenesis (column 3). Not shown are the modified inputs with removal of island isolation in Indomalaya, as this modification operates on cost distances between grid cells in Indomalaya rather than being physically manifested in the landscape. Mean temperature (°C), standard deviation (SD) of temperature, approximate area (grid cells), and relative proportion of fragments (disconnected sites) are shown.

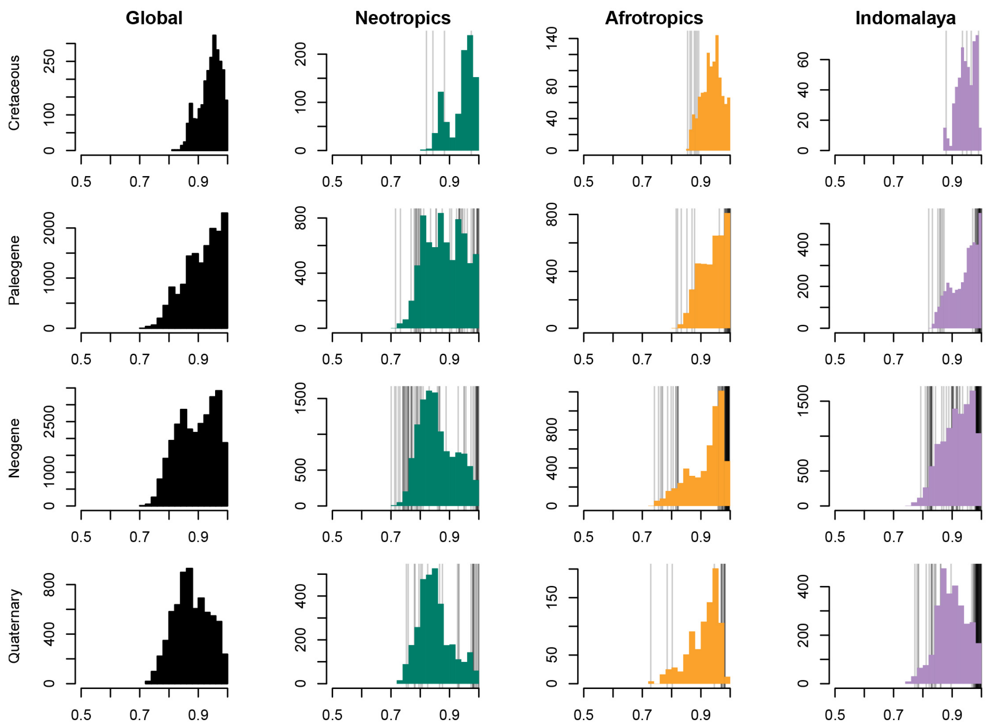

Fig. S8. Cumulative trait density of global and Neotropical, Afrotropical and Indomalayan tropical moist forest (TMF) communities for geological periods (i.e. Cretaceous [110–65 Ma], Paleogene [65–23 Ma], Neogene [23–2.6 Ma] and Quaternary [2.6–0 Ma]). Vertical transparent lines (alpha=0.2) are drawn for each extinction event during the plotted time period. Trait density is calculated from the mean species temperature-niche traits (i.e. mean of the standardised temperature niche position after the selection process of all populations (n) for all species $\overline{T} = \frac{T_1+T_2+\cdots+T_n}{n}$) accumulated over each time period. Temperature values ranging from -47 to 28 are standardized here to be between 0 and 1.

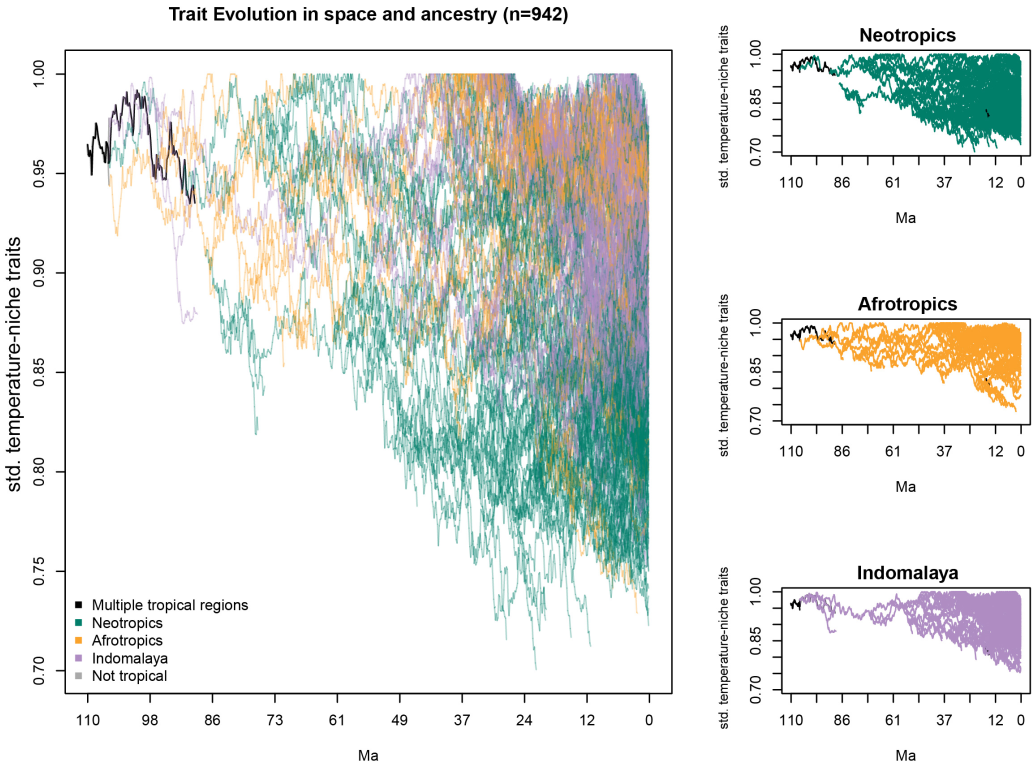

Fig. S9. Evolution of species' mean temperature-niche traits over time for all species of one simulation (matching Fig. 1C in the main text) for the entire globe (left-panel) and each tropical moist forest region (right panels), with ancestor lineages shown in black. Splitting lines indicate speciation events and lines finishing before the present indicate extinction events. The mean temperature-niche trait is calculated over all populations (n) of each species separately $\overline{T} = \frac{T_1+T_2+\cdots+T_n}{n}$). Temperature values ranging from -47 to 28 are standardized here to be between 0 and 1.

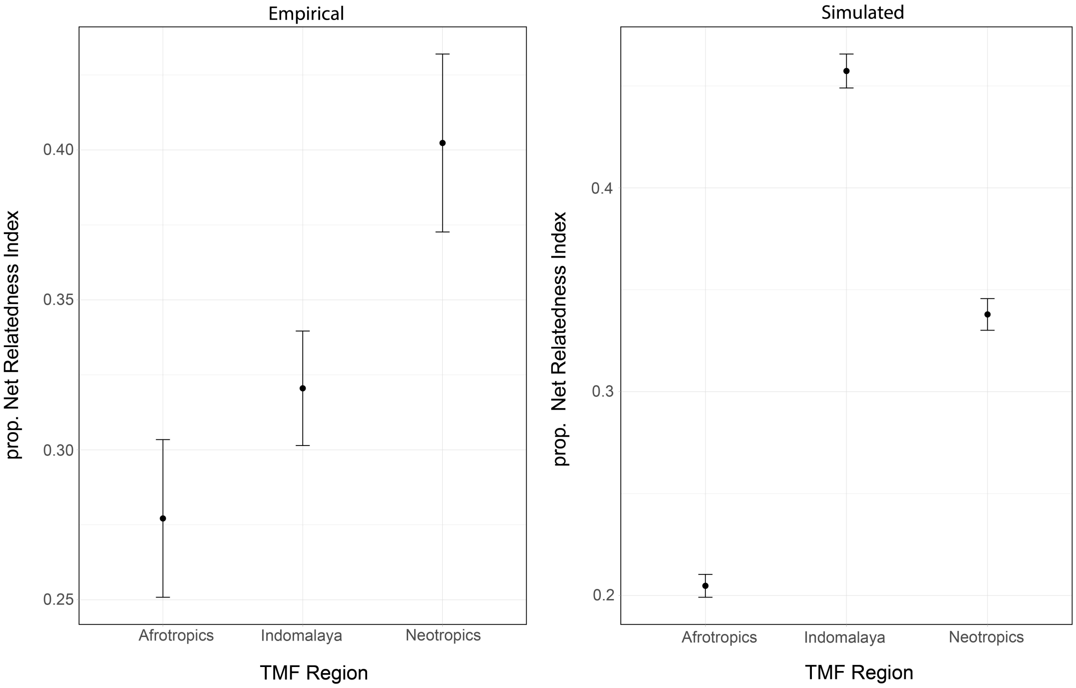

Fig. S10. Relative proportion of the net relatedness index (NRI) in Neotropical, Afrotropical and Indomalayan tropical moist forests (TMF) for empirical data from terrestrial vertebrate clades and simulated data from models generating the pantropical diversity disparity pattern. Larger values indicate greater phylogenetic clustering. The Afrotropical TMF biome is less clustered in empirical and simulated data than the Neotropical and Indomalayan TMF regions.

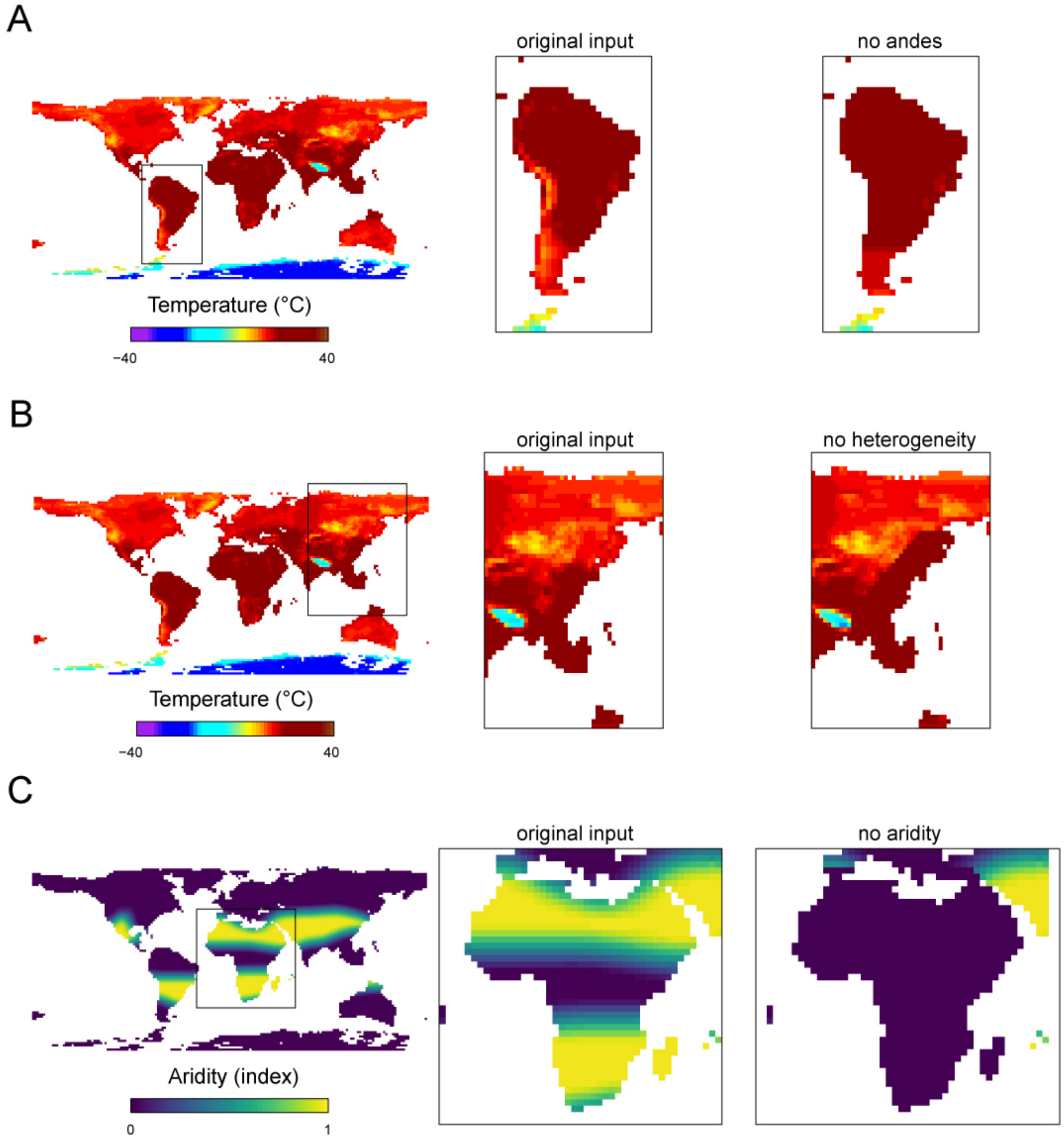

Fig. S11. Simulation input landscape modifications from original world map at 30 Ma (left column) and specific modified regions (middle and right column): (A) Removal of the Andes leading to homogenization of temperatures on South America; (B) Homogenization of temperatures in Indomalaya; (C) Removal of aridity in the African continent.

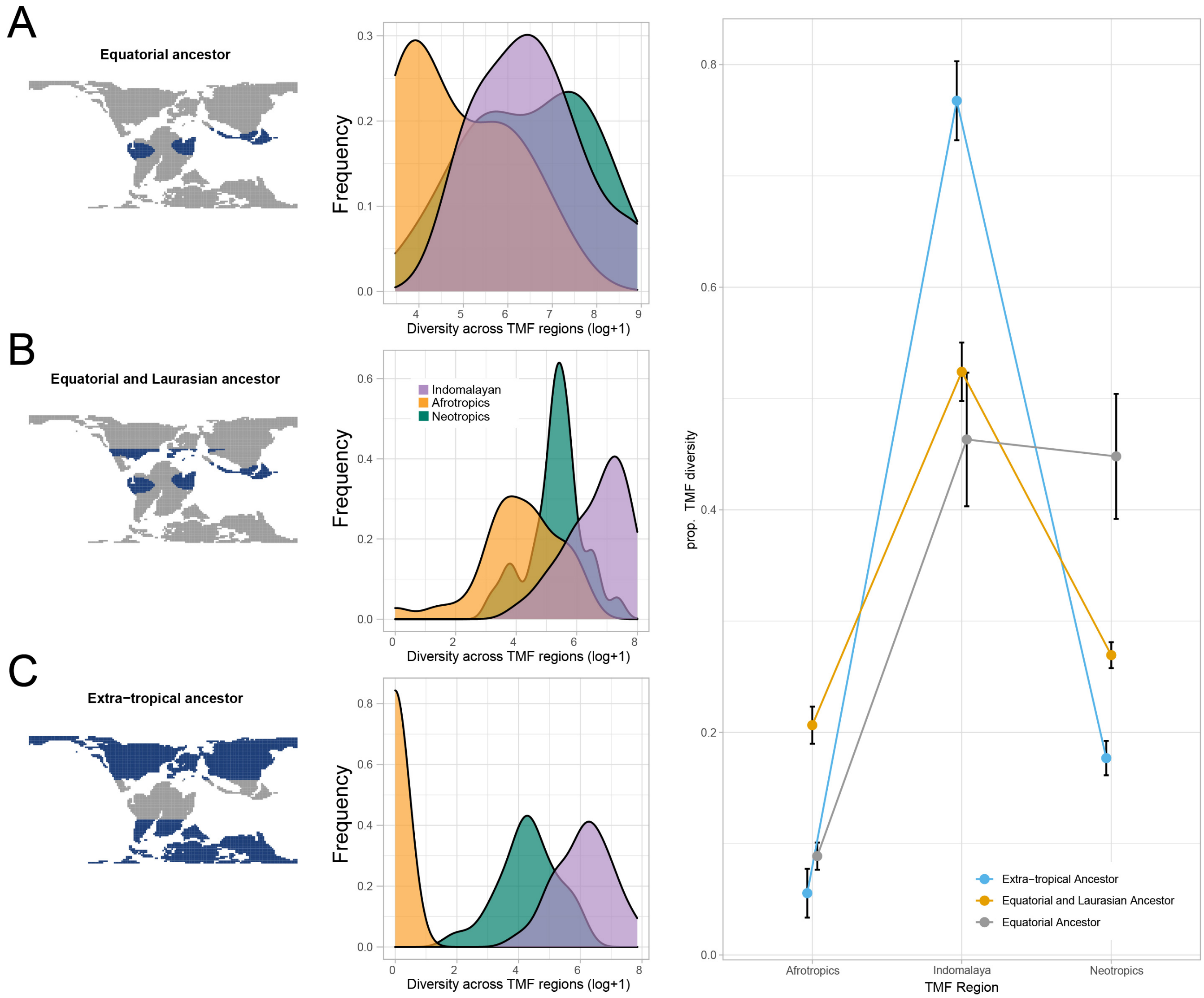

Fig. S12. Different simulation initialisation scenarios for the location of the ancestor species: (A) Locations of ancestor species at the beginning of the simulation 110 Ma (from top to bottom: original equatorial initial conditions, Laurasian ancestor, and an exclusively extra-tropical ancestor); (B) Simulated diversity across three tropical moist forest (TMF) regions under each initialisation scenario; (C) Mean proportion of diversity in each TMF region under each initialisation scenario.

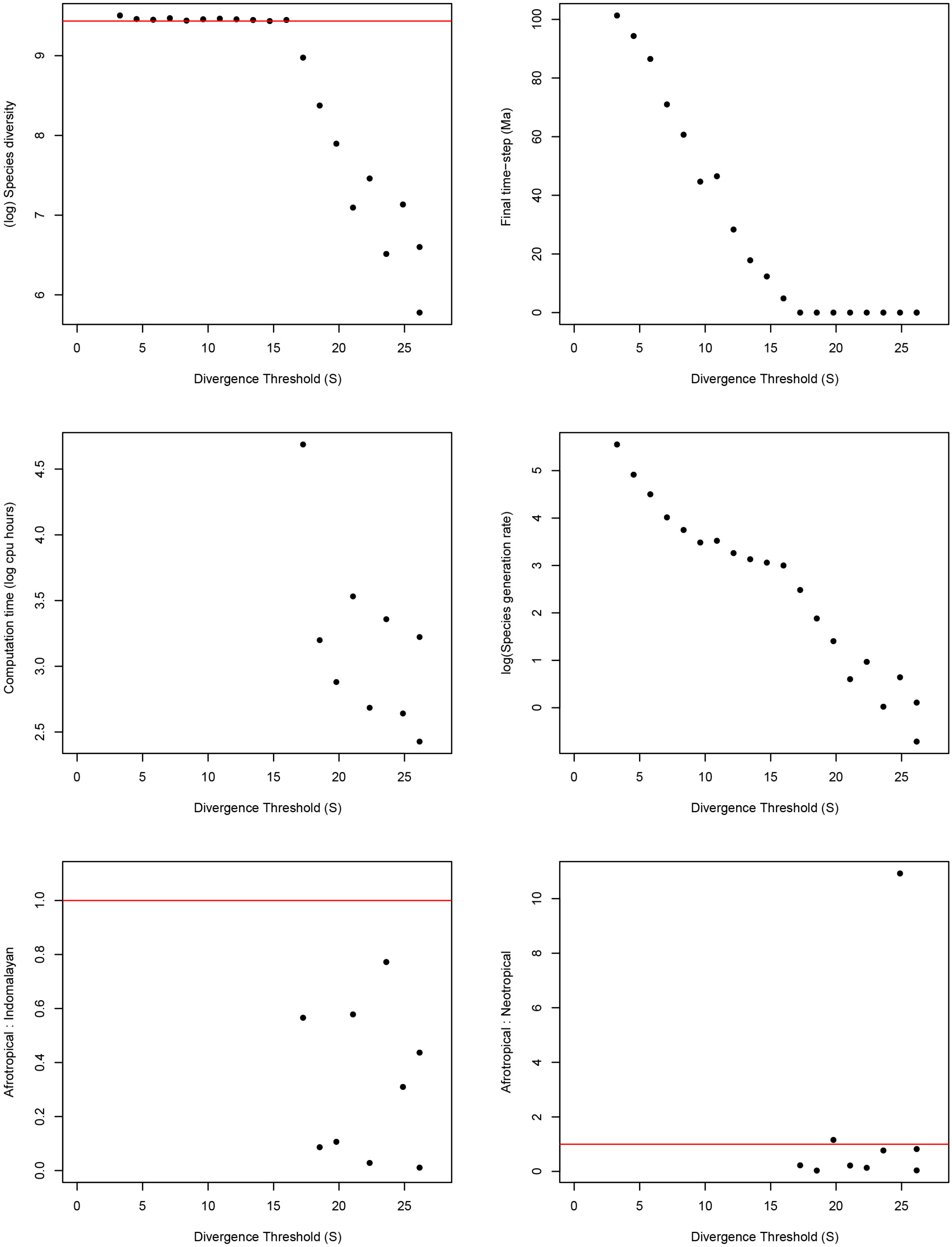

Fig. S13. Relationship between the divergence threshold parameter (S) and species diversity (top left panel; the red line indicates the maximum species threshold of 12,500). Shown are the final time-step (Ma; top-right panel), log computation time (hours, middle-left panel), log speciation generation rate (middle-right panel), ratio of Afrotropical to Indomalayan tropical moist forest diversity (bottom-left panel; red line indicates the 1:1 ratio), and the ratio of Afrotropical to Neotropical moist forest diversity (bottom-right panel; red line indicates the 1:1 ratio values above the red line have greater diversity in the Afrotropics).

Movie S1. The origins of species richness with the gen3sis model of species diversification across 110 Myr of paleoenvironmental reconstructions. This animation is an example of the model dynamics using one simulation which generated the observed disparity in pantropical diversity.

Movie S2. Evolutionary dynamics of traits by region. (A) Phylogeny with mean temperature-niche-trait values and coloured by region (i.e. more than 50% of a species distribution is in one region; if not, species are considered to belong to multiple regions). Extinction events are shown as crosses in the moving timeline, with colours corresponding to regions with the most events. The number of extant species is updated in the next time-step depending on the previous speciation and extinction events. (B) Temperature-niche-trait frequency distribution through time of all extant species in the simulation (global), as well as in the Neotropical, Afrotropical and Indomalayan regions separately. Dashed vertical lines show the mean trait value of each region or the unique trait value if only one species occurs.

Movie S3. Tropical zone dynamics. The estimated location of megathermal climate in each of three biogeographic regions (Afrotropics, Neotropics, Indomalaya) based on reconstructions of temperature and aridity.

SI Dataset S1 (Dataset_S1.csv)

Distribution of species diversity in plant and vertebrate clades. Total clade level diversity and species diversity in tropical moist forests (TMF) across the Neotropics, Indomalaya and Afrotropics. Pantropical clades are found in all three TMF regions with at least one-third of the clades' total diversity spread throughout these regions. Pantropical diversity disparity (PDD) clades show lower diversity in TMF in the Afrotropics than in the Neotropics and Indomalaya.

SI Dataset S2 (Dataset_S2.csv)

Environmental and species richness data across 110 km x 110 km grid cells in Neotropical, Indomalayan and Afrotropical moist forest sites. Variables include x and y coordinates in the Behrmann equal area coordinate reference system, potential evapotranspiration (PET), mean annual temperature (MAT), mean annual precipitation (MAP), amphibian, mammal, bird and squamate reptile species richness and biogeographic region, as well as the first two principal components of a principal component analysis on PET, MAT and MAP (PC1, PC2).

SI Dataset S3 (Dataset_S3.csv)

Global reconstructed paleo-temperature estimates and spatial coordinates across 200 million years at 170,000 year intervals at 2° spatial resolution.

SI Dataset S4 (Dataset_S4.csv)

Gen3sis model parameters and biodiversity summary statistics. Summary statistics include the number of extant species, the number of extinct species, the total number of species, the number of species within the tropical moist forest biome boundaries in the Neotropics, the Afrotropics and Indomalaya, the pantropical index, and the pantropical disparity index, as well as the running time-step and diversity of unfinished simulations.

SI Dataset S5 (Dataset_S5.csv)

Net relatedness index (NRI) values for vertebrate clades showing an observed disparity in pantropical diversity in the Neotropical, Indomalayan and Afrotropical moist forest regions and associated *P*-values. Positive values indicate phylogenetic clustering, whereas negative values indicate phylogenetic overdispersion.

References

1. O Hagen, et al., Mountain building, climate cooling and the richness of cold-adapted plants in the northern hemisphere. *J. Biogeogr.* **46** (2019).
2. CR Scotese, N Wright, Paleomap paleodigital elevation models (paleodems) for the phanerozoic (2018).
3. CR Scotese, H Song, BJ Mills, DG van der Meer, Phanerozoic paleotemperatures: The earth's changing climate during the last 540 million years. *Earth-Science Rev.* **215**, 103503 (2021).
4. AJ Boucot, C Xu, CR Scotese, RJ Morley, *Phanerozoic paleoclimate: an atlas of lithologic indicators of climate*, Concepts in Sedimentology and Paleontology. (Society of Economic Paleontologists and Mineralogists (Society for Sedimentary Geology), Tulsa, U.S.A.), (2013).
5. CR Scotese, Some thoughts on global climate change: the transition from icehouse to hothouse. *Paleomap project* **21**, 1 (2) (2015).
6. DL Royer, RA Berner, IP Montañez, NJ Tabor, DJ Beerling, Co2 as a primary driver of phanerozoic climate. *GSA Today* **14** (2004).
7. SE Fick, RJ Hijmans, Worldclim 2: new 1-km spatial resolution climate surfaces for global land areas. *Int. J. Climatol.* **37**, 4302–4315 (2017).
8. J van Etten, R package gdistance: Distances and routes on geographical grids. *J. Stat. Softw.* **76** (2017).

9. O Hagen, et al., Gen3sis: the general engine for eco-evolutionary simulations on the origins of biodiversity. *bioRxiv* **0** (2020).
10. V Grimm, et al., A standard protocol for describing individual-based and agent-based models. *Ecol. Model.* **198**, 115–126 (2006).
11. S Kucherenko, D Albrecht, A Saltelli, Exploring multi-dimensional spaces: A comparison of latin hypercube and quasi monte carlo sampling techniques. *arXiv* **1** (2015).
12. J Wible, G Rougier, M Novacek, et al., Cretaceous eutherians and laurasian origin for placental mammals near the k/t boundary. *Nature* **447**, 1003–1006 (2007).
13. J Coyne, H Orr, *Speciation.* (Sinauer), (2004).
14. D Curnoe, A Thorne, JA Coate, Timing and tempo of primate speciation. *J. Evol. Biol.* **19**, 59–65 (2006).
15. RS Etienne, H Morlon, A Lambert, Estimating the duration of speciation from phylogenies. *Evolution* **68**, 2430–2440 (2014).
