# Supplementary figures and images for: Earth history events shaped the evolution of uneven biodiversity across tropical moist forests
Source: Proc Natl Acad Sci U S A. 2021 Oct 1;118(40):e2026347118. doi: 10.1073/pnas.2026347118 (PMC8501849; doi:10.1073/pnas.2026347118)

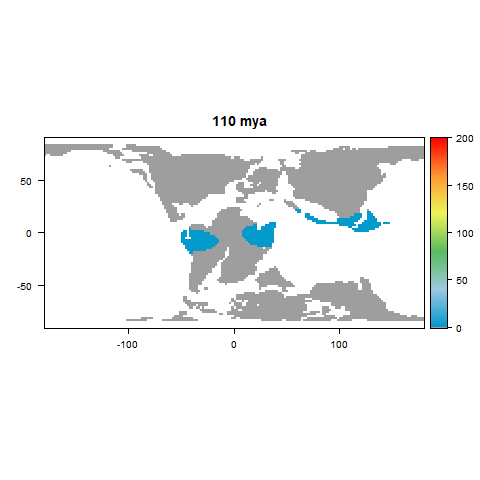

Supplement: Supplementary File [file pnas.2026347118.sm01.gif]

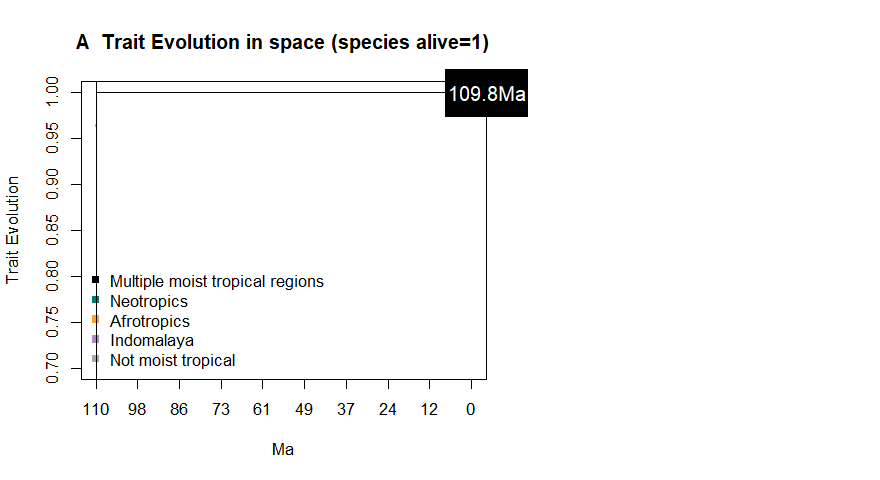

Supplement: Supplementary File [file pnas.2026347118.sm02.gif]

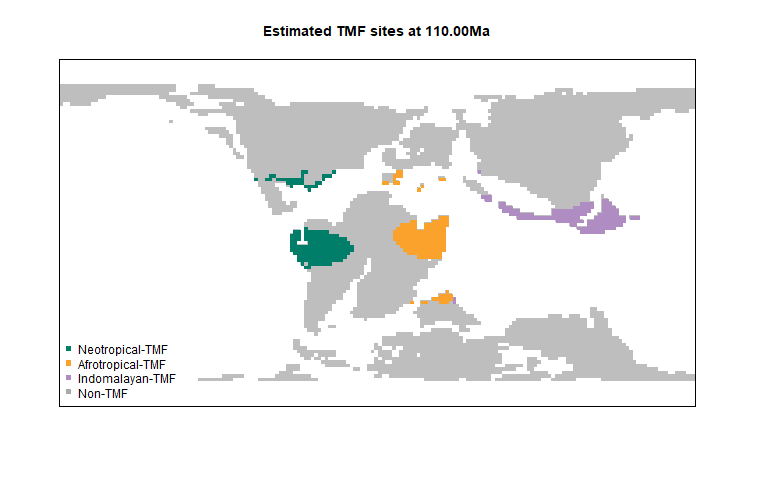

Supplement: Supplementary File [file pnas.2026347118.sm03.gif]
